# Supplementary material for: Transient neurological symptoms in the older population: report of a prospective cohort study—the Medical Research Council Cognitive Function and Ageing Study (CFAS)
Source: BMJ Open. 2013 Jul 23;3(7):e003195. doi: 10.1136/bmjopen-2013-003195 (PMC3731761; doi:10.1136/bmjopen-2013-003195)
Supplement: Author's manuscript [file bmjopen-2013-003195.draft_revisions.pdf]

Transient neurological symptoms in the older population: Report of a prospective cohort study - the Medical Research Council Cognitive Function and Ageing (CFAS) Study

Journal:	*BMJ Open*
Manuscript ID:	bmjopen-2013-003195
Article Type:	Research
Date Submitted by the Author:	09-May-2013
Complete List of Authors:	Mavaddat, Nahal; University of Cambridge, Department of Public Health and Primary Care<br>Savva, George; Trinity College Dublin, Medical Gerontology<br>Lasserson, Daniel; University of Oxford, Primary Care Health Sciences<br>giles, matthew; Biomedical Research Centre, John Radcliffe Hospital, Stroke Prevention Research Unit,<br>Brayne, Carol; University of Cambridge, Institute of Public Health<br>Mant, Jonathan; University of Cambridge, General Practice and Primary Care Research Unit
**Primary Subject Heading**:	Neurology
Secondary Subject Heading:	Cardiovascular medicine, Epidemiology
Keywords:	Stroke < NEUROLOGY, EPIDEMIOLOGY, Stroke medicine < INTERNAL MEDICINE

SCHOLARONE™
Manuscripts

Transient neurological symptoms in the older population: Report of a prospective cohort study - the Medical Research Council Cognitive Function and Ageing Study (CFAS)

Nahal Mavaddat, George M Savva, Daniel Lasserson, Matthew Giles, Carol Brayne, Jonathan Mant

Department of Public Health and Primary Care, University of Cambridge, Strangeways Laboratory, Worts Causeway, Cambridge, UK CB1 8RN Nahal Mavaddat clinical lecturer Jonathan Mant professor The Irish Longitudinal Study on Ageing, Department of Gerontology, Trinity College Dublin, Lincoln Gate, Dublin 2, Republic of Ireland George Savva lecturer Department of Primary Care Health Sciences, University of Oxford, Oxford, U.K. New Radcliffe House, 2nd floor, Walton Street, Jericho, UK OX2 6NW Daniel Lasserson clinical lecturer Stroke Prevention Research Unit, NIHR Biomedical Research Centre, Oxford University, Department of Clinical Neurology, John Radcliffe Hospital, Oxford, UK OX3 9DU Matthew Giles consultant physician
Department of Public Health and Primary Care, University of Cambridge, Institute of Public Health, Forvie Site, Robinson Way Causeway, Cambridge, UK CB2 0SR Carol Brayne professor

Correspondence to: Nahal Mavaddat nm212@medschl.cam.ac.uk

The Corresponding Author has the right to grant on behalf of all authors and does grant on behalf of all authors, an exclusive licence (or non exclusive for government employees) on a worldwide basis to the BMJ Publishing Group Ltd to permit this article (if accepted) to be published in BMJ editions and any other BMJPGL products and sublicences such use and exploit all subsidiary rights, as set out in our licence.

For peer review only

Article Summary

Article Focus:

- Prompt initiation of secondary prevention following TIA is associated with up to 80% reduction in risk of subsequent stroke.
- Many people presenting to specialist TIA clinics with transient neurological symptoms do not have TIA.
- The prevalence and incidence of transient neurological symptoms (as opposed to TIA) in older age groups is unknown.

Key Messages:

- In a large multi-centred community based study representative of the older population in the UK we found a high prevalence and incidence of transient neurological symptoms, significantly greater than that of TIAs.
- This highlights the need for adequate provision of TIA services and better diagnostic tools, especially as the public becomes more sensitised to possible cerebrovascular symptoms, and primary care practitioners more aware of the potential benefits of rapid response to suspected TIA.

Strengths and Limitations of this study:

- Strengths - Previous studies of incidence and prevalence of transient neurological symptoms have mostly been conducted in populations not representative of older age groups where TIA is most common and few have been undertaken in UK populations.

For peer review only

- Limitations - The incidence of transient neurological symptoms in the study was determined in people without severe cognitive impairment, and may be an underestimate since cognitive impairment can be a manifestation of vascular disease. The wording of questions to identify transient neurological symptoms may have picked up some people who had symptoms lasting more than 24 hours. Such patients are nevertheless likely to draw on TIA services for further assessment.

For peer review only

ABSTRACT

Objective: Transient ischaemic attack (TIA) is a recognised risk factor for stroke in the older population requiring timely assessment and treatment by a specialist. The need for such TIA services is driven by the epidemiology of transient neurological symptoms, which may not be caused by TIA. We report prevalence and incidence of transient neurological symptoms in a large UK cohort study of older people. **Design** Longitudinal cohort study **Setting and Participants:** The MRC Cognitive Function and Aging Study (CFAS) is a population representative study derived from random samples of people in their 65th year. Interview at baseline included questions about stroke and transient neurological symptoms, repeated in a sub-sample after two years. Patients were flagged for mortality. **Main Outcome Measures** Prevalence and two year incidence of transient neurological symptoms. **Results** In 11,903 participants without history of stroke, 271(2.3%) reported transient problems with speech, 872 (7.6%) with sight, and 596 (5.1%) weakness in a limb, with 1456 (12.7%) reporting at least one symptom. Of those re-interviewed (n=6748), 675 (9.8%) reported at least one symptom over two years. **Conclusions** Lifetime prevalence and incidence of transient neurological symptoms in people 65 and over is substantially higher than incidence of TIA in hospital and population-based studies. With increasing public education regarding stroke, TIA services may need to cope with an increase in demand as a greater proportion of people with transient neurological symptoms present for urgent assessment.

For peer review only

INTRODUCTION

Transient ischaemic attack (TIA) is an established and powerful risk factor for stroke. 8% of patients who have a TIA suffer a stroke within 7 days, many within 48 hours.[1;2] Immediate specialist assessment and treatment is associated with substantial reductions in this early risk of stroke, [3] and is recommended for people with suspected TIA. [4]

As awareness grows of the urgency of early management of TIA amongst both primary care practitioners and the general public, [5] more patients with symptoms suggestive of a TIA are likely to present to primary and secondary care services. Need for specialist services will be driven by incidence of symptoms that might represent TIA (i.e. transient neurological symptoms) rather than the epidemiology of TIA *per se*. Currently about 48,000 probable or definite TIAs (transient neurological symptoms lasting less than 24 hours of likely vascular aetiology), and 43,000 minor strokes (stroke events causing minimal or no neurological deficits) are managed as outpatients every year in England, but it is not clear to what extent this might be the tip of the iceberg. [6] Data are available worldwide on the prevalence and incidence of TIA. [7-11] The epidemiology in the community of transient neurological symptoms (i.e. neurological symptoms of sudden onset of which TIA is a subset), is however less well defined. Previous studies of incidence and prevalence of transient neurological symptoms, have mostly been conducted in populations not representative of older age groups where TIA is most common. [7;11-16] Very few have been undertaken in UK populations. While incidence of transient neurological symptoms will drive need for

For peer review only - http://bmjopen.bmj.com/site/about/guidelines.xhtml

specialist TIA services, prevalence of transient neurological symptoms in the community is also important. TIA is associated with a long term increase in risk of stroke, [17;18] so there may be value in diagnosing 'old' events that have not presented to medical services in order to target secondary prevention. Hence the need to know both the incidence and prevalence of transient neurological symptoms in the community.

This study reports prevalence and incidence of three common transient neurological symptoms (limb weakness, loss of speech and disturbance of vision) in a population based multi-centred cohort study in the UK (England and Wales) in those aged 65 and over, the Medical Research Council Cognitive Function and Ageing Study (CFAS). [19]

METHODS

The Medical Research Council CFAS is a population based study of individuals aged 65 years and over living in the community. The study began in 1991 and was designed to determine the incidence of dementia in the older population. It has six centres across England and Wales chosen to represent the national variation of urban-rural mix, socio-economic deprivation and rates of chronic disease.[19] Five of these with identical study designs (Oxford, Nottingham, Cambridgeshire Gwynedd and Newcastle) are used in the present investigation. The sixth centre (Liverpool) used a different design and is therefore excluded. Random samples of people in their 65th year and above were obtained from Family Health Service Authority lists (agency responsible for maintaining registers of general practice populations at that time). The sample was stratified by age (65-74, and 75 years and over) and equal numbers were randomly selected from these groups with the aim of recruiting 2500 to each centre.

For peer review only

All study centres obtained ethical approval from local research committees. (REC Ref: 05/MRE05/37). Full details of methodology are available elsewhere. [19]

Eligible participants provided informed consent (or their proxies where appropriate). Trained interviewers undertook baseline interviews in the participants' homes including socio-demographic characteristics, cognitive function and disease history including previous stroke, coronary heart disease and diabetes (full details at www.cfas.ac.uk). Based on baseline screening, the study sample was divided into two groups at baseline: people without cognitive impairment and a group consisting of those with cognitive impairment plus a stratified subsample of those without cognitive impairment. The first group underwent no further assessment at baseline and were 'rescreened' after two years. The second group underwent a further detailed cognitive assessment at baseline, but were not followed up for transient neurological symptoms. The estimates of prevalence of transient neurological symptoms are calculated for all people at baseline, while the incidence estimates use only the first group. All participants were flagged with the NHS Central Register. Deaths and underlying causes of death attributed to stroke (ICD codes 430-438) were notified to the study.

Prevalence of transient neurological symptoms

At baseline all participants were asked "Have you ever experienced sudden problems with:

(1) speech, which got better after a day?

(2) weakness in the arms or legs, which got better after a day?

(3) sight, which got better after a day?"

For peer review only

Social class was determined using the Registrar General's Occupational Classification. Cognitive status was determined using the Mini Mental State Examination (MMSE), [20] and the Automated Geriatric Examination for Computer Assisted Taxonomy (AGECAT). [21]

Incidence of transient neurological symptoms

All those in the 'rescreen' arm were asked at two year follow-up if they had experienced each transient neurological symptom in the past two years.

Analysis

All analyses were carried out using STATA version 11. Inverse probability weights were used throughout to ensure the sample was representative of the target population. Weights were estimated with logistic regression using presence in each phase of the study as an outcome and taking into account over-sampling of over-75s at baseline. Weights for incidence calculations adjust for attrition based on baseline characteristics and stratified selection into the assessment arm as appropriate. Baseline prevalence of each transient neurological symptom was calculated for age specific strata and as a weighted percentage to provide a population estimate of prevalence for people aged 65 and over. Associations with gender, age, social class (manual (IIIb, IV, V) and non-manual (I, II, IIIa)), and cardiovascular comorbidity were explored using logistic regression models adjusting for all other factors and cognitive function based on MMSE by score (less than 18, 18-21, 22-25 and 26-30).

Two year incidence of transient neurological symptoms was estimated using weighted percentages of those reporting any of the symptoms during follow-up. Weights were adjusted for refusals, drop-outs and for non-reassessment of people with cognitive

For peer review only - http://bmjopen.bmj.com/site/about/guidelines.xhtml

impairment. Calculation of attrition weights for incident transient neurological symptoms excluded those who died between baseline and follow up. These estimates are therefore applicable to the population over 65 without severe cognitive impairment or stroke at baseline surviving at least two years.

RESULTS

The participation rate in the CFAS study was 80% (13,004/16,258). For the prevalence analysis, participants who had a stroke at baseline (963, 7.4%) or for whom baseline information about stroke was missing (138, 1.1%) were excluded, leaving 11903 participants. 2,283 of the original participants were not allocated to the 'rescreen' arm at two years; 754 died, 1973 declined to participate and 145 were lost to follow-up, leaving 6,748 (76% response rate of potential participants) for the incidence analysis.

Table 1 shows the demographic features of participants in the CFAS studied in this analysis at baseline. 40% of participants were male and 60% of participants were female. 12.3% of all participants reported at least one transient neurological symptom. 2.3% reported transient loss of speech, 5.1% transient weakness and 7.6% transient loss of sight.

Table 2 shows prevalence data with weighted percentages and odds ratios (adjusted for age, sex, social class, cognition and cardiovascular morbidities) of reported transient neurological symptoms at baseline by demographic factors and comorbidities. 12.8% of males and 12.0% of females reported at least one of the three transient neurological symptoms of loss of speech, loss of sight, or weakness. There was no significant association of gender with reporting at least one transient

For peer review only - http://bmjopen.bmj.com/site/about/guidelines.xhtml

neurological symptom, but a significantly lower odds of reporting a transient neurological symptom in the over 85 age group. Problems of memory and recall may have contributed to under-reporting in this group. Greater rates of mortality associated with neurological incidents in the oldest old population may also lead to under estimation of rates of transient neurological symptoms. The odds of reporting any transient neurological symptom were higher in those in manual compared to non-manual social classes. The presence of cardiovascular morbidities of angina and heart attack were significantly associated with the odds of having at least one of the transient neurological symptoms.

There was no difference between genders with regards to reporting individual transient neurological symptoms of loss of speech, loss of sight or weakness. However, there was a significantly lower odds of reporting transient visual loss in those over 85. This may partly explain the overall lower prevalence of reporting at least one transient neurological symptom in this group. The cause for this is unclear, however in addition to problems of recall, may in part relate to the greater likelihood of chronic visual problems potentially masking transient visual losses in the oldest old population. There was an increased odds of reporting transient symptoms of weakness and loss of sight in manual compared to non-manual social classes. The presence of cardiovascular co-morbidities of angina and heart attack were associated with higher odds of all three symptoms.

Table 3 shows two year incidence of transient neurological symptoms in respondents attending the “rescreen”. 9.8% of all participants reported at least one transient neurological symptom over the 2 years, with the highest incidence of reported

For peer review only

transient neurological symptom being due to loss of sight followed by weakness, with loss of speech being the least frequently reported transient neurological symptom. The incidence of each of the transient neurological symptoms was highest in the 75 to 84 age group and lowest in the over 85 age group.

DISCUSSION

Our findings suggest that transient neurological symptoms are common in the older population in England and Wales, with at least 12% of people aged 65 and over having experienced a transient neurological symptom of the arm or leg, speech or vision better after a day, and approximately 5% experiencing at least one such symptom over the course of a year. The commonest of these was transient symptoms of vision, followed by limb weakness. Incidence of at least one transient neurological symptom in the CFAS population was approximately 2.6 times greater than that of confirmed TIA in the Oxford Vascular Study (OxVASC) in people aged over 85 (approximately 15 per 1000 per year), 8 times greater in those aged 75 to 84 (7 per 1000 per year), and approximately 15 times greater in those aged 65 to 74 (3 per 1000 population per year). [10]

The prevalence and incidence of transient neurological symptoms in the CFAS was somewhat higher than those found by questionnaires used in other studies internationally (mostly conducted in younger age groups), but comparable to those of Wilkinson et al. in an over 60 age group in a US based study. [7;12-14;16] Table 4 (online) shows the prevalence of sudden onset of neurological symptoms of weakness in a limb or of loss of speech or sight in previous population and community studies. [7;12-

For peer review only

[14;16] Questions used to elicit transient neurological symptoms in the CFAS are likely to have captured greater numbers of transient neurological events in the population compared to those seeking more specific vascular symptoms, or to the few studies defining more precisely the onset, offset and timing of the event. [7;12-14] While some respondents will have been describing true transient ischaemic events, it is likely that many did not have a true TIA. Validated measures to determine the presence of previous transient ischaemic attack are limited, and most studies of transient neurological symptoms in questionnaires overestimate true transient ischaemic attacks. [13;14;22] Wilkinson et al. (1979) suggest that around 10% of transient symptoms reported in a questionnaire are subsequently diagnosed as TIA after neurologist assessment.[14] Self-reported transient neurological symptoms in other community based studies have low positive predictive value, with higher values only in studies in outpatient populations (over 70%). [13;14;22-24] This suggests that the use of screening to identify possible past transient ischaemic attacks may generate unnecessary extra strain on primary care and secondary TIA services with limited benefit.

Patients presenting with transient neurological symptoms present considerable diagnostic dilemmas for primary care practitioners reflected in low rates of TIA diagnosis confirmation in patients referred to UK TIA clinics (over 50% being for non-TIA causes). [25-27] Many symptoms of TIA are non-specific and occur in non-vascular syndromes. Dizziness, ophthalmological problems, migraine, epilepsy, nerve entrapment or psychological states are the commonest non-TIA diagnoses. [25;26;28-30] Often, however, no diagnosis can be determined. [25;26] Scales such as the ABCD2 [2] help determine the urgency of TIA referral, but do not distinguish TIA from non-TIA symptoms. Wilkinson et al. reported transient loss of speech and loss of sight to be

For peer review only - http://bmjopen.bmj.com/site/about/guidelines.xhtml

more reliable than limb weakness for neurologist diagnosis of TIA, [14] while Hart et al. found the strongest and most consistent relationship with subsequent stroke to be loss of power in an arm.[7] Other studies suggest that the presence of certain symptoms such as headache, dizziness, loss of consciousness, memory loss, blurred vision, generalised weakness, pain in limbs and seizures make the diagnosis of TIA less likely. [14;30;31]

Study limitations

The incidence of transient neurological symptoms in the CFAS was determined in people without severe cognitive impairment, and may be an underestimate since cognitive impairment can be a manifestation of vascular disease. [32;33] Some symptoms of TIA (e.g. posterior circulation or pure sensory symptoms) were not sought in the CFAS. The wording of questions to identify transient neurological symptoms may have picked up some people who had symptoms lasting more than 24 hours. Such patients are nevertheless likely to draw on TIA services for further assessment. In addition, it was not possible to determine whether different transient neurological symptoms in a participant occurred simultaneously or separately in time. The problem of recall bias in these self-reports of transient symptoms needs to be considered. Finally, baseline and two year follow-up were carried out in the 1990s and changes in rates of vascular events may have occurred since then. While the age-specific incidence of stroke appears to be declining, it is not clear that the same is true of TIA. [10;34;35] As well, this study is more recent than many studies estimating rates of transient neurological symptoms in the population, many of which have been carried out during the 1970’s or ‘80’s. [12-14]

For peer review only

Conclusion

In a large multi-centred community based study highly representative of the older population in the UK and conducted in the age group where TIAs are most common, we found a high prevalence and incidence of transient neurological symptoms. The incidence of such symptoms in the community is significantly greater than that of TIAs, suggesting that potential demand for TIA clinics where a confirmation of TIA diagnosis is made by a specialist, might rise significantly in the future as the public becomes more sensitised to possible cerebrovascular symptoms, and primary care practitioners more aware of the potential benefits of rapid response to suspected TIA. This highlights the need for adequate provision of TIA services and the potential importance of the development of valid diagnostic tools to assist the general practitioner and hospital doctor in better triage of people presenting with transient neurological symptoms.

ACKNOWLEDGEMENTS

We thank the MRC CFAS study group for data collection and management. We are also grateful to all respondents, their families and their primary care teams from across the country for their participation in the CFAS.

COMPETING INTERESTS

"All authors have completed the ICMJE uniform disclosure form at www.icmje.org/coi_disclosure.pdf and declare: no support from any organisation for the submitted work; no financial relationships with any organisations that might have

For peer review only

an interest in the submitted work in the previous three years; no other relationships or activities that could appear to have influenced the submitted work."

FUNDING

The MRC CFAS Study has had funding by major awards from the Medical Research Council (MRC/G9901400) and Department of Health. NM was funded by a National Institute for Health Research (NIHR) Walport Clinical Lectureship in Primary Care Research. None of the sponsors influenced the design or conduct of the study or the analysis or interpretation of the findings. All researchers acted independently from funders in the design and carrying out of this study.

AUTHORS CONTRIBUTIONS

CB is Principal Investigator of the MRC CFAS Study. NM and GS contributed to analysis of data. All authors contributed to study design and intellectual input. All authors, external and internal, had full access to all of the data (including statistical reports and tables) and can take responsibility for the integrity of the data and accuracy of the data analysis.

DATA SHARING

Data sharing through application.

For peer review only

Reference List

(1) Coull AJ, Lovett JK, Rothwell PM. Population based study of early risk of stroke after transient ischaemic attack or minor stroke: implications for public education and organisation of services. *BMJ* 2004; 328(7435):326.

(2) Rothwell PM, Giles MF, Flossmann E, Lovelock CE, Redgrave JN, Warlow CP et al. A simple score (ABCD) to identify individuals at high early risk of stroke after transient ischaemic attack. *Lancet* 2005; 366(9479):29-36.

(3) Rothwell PM, Giles MF, Chandratheva A, Marquardt L, Geraghty O, Redgrave JN et al. Effect of urgent treatment of transient ischaemic attack and minor stroke on early recurrent stroke (EXPRESS study): a prospective population-based sequential comparison. *Lancet* 2007; 370(9596):1432-1442.

(4) Department of Health . Department of Health. National Stroke Strategy. 7.

(5) Department of Health. Stroke: Act F.A.S.T. awareness campaign. 2009. Department of Health.

(6) Giles MF, Rothwell PM. Substantial underestimation of the need for outpatient services for TIA and minor stroke. *Age Ageing* 2007; 36(6):676-680.

(7) Hart CL, Hole DJ, Smith GD. The relation between questions indicating transient ischaemic attack and stroke in 20 years of follow up in men and women in the Renfrew/Paisley Study. *J Epidemiol Community Health* 2001; 55(9):653-656.

(8) Mant J, Wade DT, Winner S. Health care needs assessment: stroke. In: Stevens A, Raftery J, Mant J, Simpson S, editors. Health care needs assessment: the epidemiologically based needs assessment reviews. 2nd ed. Oxford: Radcliffe Medical Press; 2004. 141-244.

(9) Orlandi G, Gelli A, Fanucchi S, Tognoni G, Acerbi G, Murri L. Prevalence of stroke and transient ischaemic attack in the elderly population of an Italian rural community. *Eur J Epidemiol* 2003; 18(9):879-882.

(10) Rothwell PM, Coull AJ, Silver LE, Fairhead JF, Giles MF, Lovelock CE et al. Population-based study of event-rate, incidence, case fatality, and mortality for all acute vascular events in all arterial territories (Oxford Vascular Study). *Lancet* 2005; 366(9499):1773-1783.

(11) Bos MJ, van Rijn MJ, Witteman JC, Hofman A, Koudstaal PJ, Breteler MM. Incidence and prognosis of transient neurological attacks. *JAMA* 2007; 298(24):2877-2885.

(12) Mules J, Henderson M, Kuller L. A population survey of symptoms suggestive of transient ischemic attacks. *Stroke* 1971; 2(2):114-127.

For peer review only

(13) Toole JF, Lefkowitz DS, Chambless LE, Wijnberg L, Paton CC, Heiss G. Self-reported transient ischemic attack and stroke symptoms: methods and baseline prevalence. The ARIC Study, 1987-1989. *Am J Epidemiol* 1996; 144(9):849-856.

(14) Wilkinson WE, Heyman A, Burch JG, Ostfeld A, Labarthe DR, Leviton A et al. Use of a self-administered questionnaire for detection of transient cerebral ischemic attacks: I. Survey of elderly persons living in retirement facilities. *Ann Neurol* 1979; 6(1):40-46.

(15) Bots ML, van der Wilk EC, Koudstaal PJ, Hofman A, Grobbee DE. Transient neurological attacks in the general population. Prevalence, risk factors, and clinical relevance. *Stroke* 1997; 28(4):768-773.

(16) Howard VJ, McClure LA, Meschia JF, Pulley L, Orr SC, Friday GH. High prevalence of stroke symptoms among persons without a diagnosis of stroke or transient ischemic attack in a general population: the REasons for Geographic And Racial Differences in Stroke (REGARDS) study. *Arch Intern Med* 2006; 166(18):1952-1958.

(17) van Wijk I, Kappelle LJ, van GJ, Koudstaal PJ, Franke CL, Vermeulen M et al. Long-term survival and vascular event risk after transient ischaemic attack or minor ischaemic stroke: a cohort study. *Lancet* 2005; 365(9477):2098-2104.

(18) Clark TG, Murphy MF, Rothwell PM. Long term risks of stroke, myocardial infarction, and vascular death in "low risk" patients with a non-recent transient ischaemic attack. *J Neurol Neurosurg Psychiatry* 2003; 74(5):577-580.

(19) Brayne C, McCracken C, Matthews FE. Cohort profile: the Medical Research Council Cognitive Function and Ageing Study (CFAS). *Int J Epidemiol* 2006; 35(5):1140-1145.

(20) Folstein MF, Folstein SE, McHugh PR. "Mini-mental state". A practical method for grading the cognitive state of patients for the clinician. *J Psychiatr Res* 1975; 12(3):189-198.

(21) Copeland JR, Dewey ME, Griffiths-Jones HM. A computerized psychiatric diagnostic system and case nomenclature for elderly subjects: GMS and AGECAT. *Psychol Med* 1986; 16(1):89-99.

(22) Lasserson DS. Initial management of suspected transient cerebral ischaemia and stroke in primary care: implications of recent research. *Postgrad Med J* 2009; 85(1006):422-427.

(23) Jones WJ, Williams LS, Meschia JF. Validating the Questionnaire for Verifying Stroke-Free Status (QVSFS) by neurological history and examination. *Stroke* 2001; 32(10):2232-2236.

(24) Wilson A, Potter J, Taub N, Moore A, Robinson T. The effectiveness of a modified version of the Wilkinson questionnaire in screening for TIA and minor stroke in the United Kingdom. *Age Ageing* 2005; 34(1):30-35.

For peer review only

(25) Murray S, Bashir K, Lees KR, Muir K, MacAlpine C, Roberts M et al. Epidemiological aspects of referral to TIA clinics in Glasgow. *Scott Med J* 2007; 52(1):4-8.

(26) Martin PJ, Young G, Enevoldson TP, Humphrey PR. Overdiagnosis of TIA and minor stroke: experience at a regional neurovascular clinic. *QJM* 1997; 90(12):759-763.

(27) Giles MF, Rothwell PM. Substantial underestimation of the need for outpatient services for TIA and minor stroke. *Age Ageing* 2007; 36(6):676-680.

(28) Boysen G, Jensen G, Schnohr P. Frequency of focal cerebral transient ischemic attacks during a 12 month period. The Copenhagen city heart study. *Stroke* 1979; 10(5):533-534.

(29) Johnson SE, Skre H. Transient cerebral ischemic attacks in the young and middle aged. A population study. *Stroke* 1986; 17(4):662-666.

(30) Amort M, Fluri F, Schafer J, Weisskopf F, Katan M, Burow A et al. Transient ischemic attack versus transient ischemic attack mimics: frequency, clinical characteristics and outcome. *Cerebrovasc Dis* 2011; 32(1):57-64.

(31) Dawson J, Lamb KE, Quinn TJ, Lees KR, Horvers M, Verrijth MJ et al. A recognition tool for transient ischaemic attack. *QJM* 2009; 102(1):43-49.

(32) De Haan EH, Nys GM, van Zandvoort MJ. Cognitive function following stroke and vascular cognitive impairment. *Curr Opin Neurol* 2006; 19(6):559-564.

(33) Selnes OA, Vinters HV. Vascular cognitive impairment. *Nat Clin Pract Neurol* 2006; 2(10):538-547.

(34) Dennis MS, Bamford JM, Sandercock PA, Warlow CP. Incidence of transient ischemic attacks in Oxfordshire, England. *Stroke* 1989; 20(3):333-339.

(35) Rothwell PM, Coull AJ, Giles MF, Howard SC, Silver LE, Bull LM et al. Change in stroke incidence, mortality, case-fatality, severity, and risk factors in Oxfordshire, UK from 1981 to 2004 (Oxford Vascular Study). *Lancet* 2004; 363(9425):1925-1933.

For peer review only

For peer review only - http://bmjopen.bmj.com/site/about/guidelines.xhtml

Table 1: Demographics of Participants in the Cognitive Function and Aging Study (CFAS) by sex, age, social class and comorbidity (Data are count (%))

	**All (n=11903)**		**Males (n=4689)**		**Females (n=7214)**	
	N	%*	N	%*	N	%*
**Age**						
65-74	5980	58.8	2635	64.5	3345	55.0
75-84	4624	32.1	1716	29.6	2908	33.8
85+	1299	9.1	338	5.9	961	11.2
**Social Class**						
I	558	4.8	225	4.8	333	4.8
II	2988	26.1	1213	26.3	1775	25.9
IIIa	1337	11.6	456	9.9	881	12.8
IIIb	4295	37.7	1865	40.8	2430	35.5
IV	1727	14.9	650	13.9	1077	15.7
V	561	4.9	196	4.3	365	5.3
**Comorbidity**						
Angina	1923	16.3	878	15.3	1045	11.1
Diabetes	672	5.5	310	6.4	362	4.9
Heart Attack	1139	9.5	632	13.6	507	6.8

*Weighted percentages

For peer review only

Table 2: Distribution and adjusted odds of transient neurological symptoms in those aged 65 or over in the Cognitive Function and Aging Study (CFAS) by sex, social class and comorbidity (Data are count (%))

	Loss of Speech			Weakness			Loss of Sight			At least one symptom		
	**N**	**%***	**OR † (95%CI)**	**N**	**%***	**OR† (95%CI)**	**N**	**%***	**OR† (95%CI)**	**N**	**%***	**OR† (95%CI)**
**Gender**												
Male	119	2.5	1.0	257	5.5	1.0	347	7.6	1.0	598	12.8	1.0
Female	152	2.2	0.9(0.7-1.2)	339	4.8	0.9(0.7-1.0)	525	7.6	1.0(0.9-1.2)	858	12.0	1.0 (0.9-1.1)
**Age**												
65-74	137	2.3	1.0	302	5.1	1.0	480	8.1	1.0	768	12.9	1.0
75-84	104	2.3	0.7(0.7-1.2)	229	5.0	0.9 (0.7-1.1)	327	7.2	0.9(0.7-1.1)	553	12.0	0.9 (0.8-1.0)
85+	30	2.5	0.8(0.5-1.3)	65	5.4	0.9(0.6-1.2)	65	5.4	0.6(0.5-0.8)	134	10.4	0.7 (0.6-0.9)
**Social Class**												
Non-manual	96	5.8	1.0	63	11.0	1.0	295	17.9	1.0	497	10.2	1.0
Manual	164	7.2	1.1 (0.9-1.5)	390	17.8	1.4 (1.2-1.7)	556	25.7	1.4(1.2-1.6)	920	14.1	1.4 (1.2-1.6)
**Comorbidity**												

For peer review only

Angina (n=1538)	64	4.3	1.5(1.0-2.2)	176	11.7	2.5(2.0-3.2)	160	10.6	1.3(1.0-1.6)	312	20.7	1.7(1.4-1.9)
Diabetes (n=672)	28	4.0	1.4(0.9-2.2)	40	6.1	1.0(0.7-1.4)	66	10.1	1.3(0.9-1.7)	102	15.2	1.1(0.9-1.4)
Heart Attack (n=1139)	52	4.7	1.8(1.2-2.7)	131	10.5	1.4(1.2-1.9)	119	11.5	1.4(1.1-1.8)	233	12.4	1.4(1.2-1.8)

* Weighted percentages

†Adjusted for age, sex, social class, cognition and cardiovascular comorbidities

‡ Reference non-manual (I,II, IIIa) compared to manual social class (IIIb,IV,V)

156 participants had missing data on the speech questionnaire, 155 on the weakness and 154 on the sight question

For peer review only

Table 3: Two year incidence of transient neurological symptoms in CFAS in those without stroke at baseline at two yr Screening Assessment, N at 2yrs= 6748 (Data are N (%))

	**Age**							
**Transient Neurological Symptom**	**All**		**65-74 N=3657**		**75-84 N=2618**		**85+ N=473**	
	**N**	**%***	**N**	**%***	**N**	**%***	**N**	**%***
Loss of Speech	149	2.1	55	1.5	84	3.3	10	2.0
Weakness	239	3.7	117	3.4	106	4.1	16	3.4
Loss of Sight	410	6.3	214	6.3	170	6.6	26	4.5
**At least one**	**675**	**9.8**	**327**	**9.3**	**304**	**11.6**	**44**	**7.8**

*Weighted percentage

98 participants had missing data on the speech questionnaire, 97 on the weakness and 105 on the sight question

For peer review only

Table 4: Epidemiological studies of self-reported prevalence of sudden neurological symptoms of weakness of limbs, loss of sight and loss of speech

Table 4a: Frequencies of self-reported sudden weakness of limbs across population studies

Study	Age Range	Date	Population	N	Question	How	Frequencies
Renfrew/ Paisley ***Hart et al. (2001)***[1]	45-64	1972-1976	General population study residents Renfrew&Paisley, Scotland Excluded those with stroke	1511 3	Have you ever without warning suddenly lost the power of an arm?	Questionnaire checked when attended a clinic	344 (2.3%)
Renfrew/ Paisley ***Hart et al. (2001)***[1]	45-64	1972-1976	General population study residents Renfrew&Paisley, Scotland Excluded those with stroke	1511 3	Have you ever without warning suddenly lost the power of a leg?	Questionnaire checked when attended a clinic	363 (2.4%)
Atherosclerosis Risk in Communities (ARIC) Cohort ***Toole et al (1996)***[2]	45-64	1987-1989	Four centres in US adults residents	12,20 5	Have you ever had sudden painless weakness on one side of your body?	Administered by researcher – questions read aloud	283 (2.3%)
Baltimore population study ***Mules et al (1971)***[3]	>40	1954-1967	Population samples – two in US	9937	Paralysis within 2 years prior to interview	Interview by trained interviewer	48(0.7%)
Reasons for geographic and racial differences in Stroke (REGARDS) ***Howard et al (2006)***[4]	> 45 (mean 65)	2003	Community dwelling individuals in US. Those with stroke and TIA excluded	1846 2	Have you ever had sudden painless weakness on one side of the body?	QVSFS questionnaire – telephone and in person	1065 (5.8%)

For peer review only

Survey of Elderly Persons Living in Retirement Facilities ***Wilkinson et al (1979)*** [5]	>60	Published 1979	Elderly living in retirement facilities in 8 cities in the US	7281	During last **12 months** any sudden occurrence of transient episode of weakness or paralysis of limbs *which subsequently cleared up completely*	Self-administered questionnaire	197 (2.7%)

For peer review only

Table 4b: Frequencies of self-reported sudden speech impairment across population studies

**Study**	**Age Range**	**Date**	**Population**	**N**	**Question**	**How**	**Frequencies**
Renfrew/ Paisley ***Hart et al. (2001)***[1]	45-64	1972-1976	General population study residents Renfrew&Paisley, Scotland Excluded those with stroke	15113	Have you ever without warning suddenly been unable to speak properly?	Questionnaire checked when attended a clinic	270 (1.8%)
Atherosclerosis Risk in Communities (ARIC) Cohort ***Toole et al (1996)*** [2]	45-64	1987-1989	Four centres in US adults residents	12,205	Have you ever had sudden episode of "speech dysfunction"?	Administered by researcher – questions read aloud	313 (2.6%)
Reasons for geographic and racial differences in Stroke (REGARDS) ***Howard et al (2006)*** [4]	> 45 (mean 65)	2003	Community dwelling individuals in US. Those with stroke and TIA excluded	18462	Have you ever had sudden loss of ability to express self verbally (or in writing)?	QVSFS questionnaire – telephone and in person	698 (3.8%)
Survey of Elderly Persons Living in Retirement Facilities ***Wilkinson et al (1979)*** [5]	>60	Published 1979	Elderly living in retirement facilities in 8 cities in the US	7281	During last **12 months** experienced any sudden loss of speech or changes in speech *which subsequently cleared up completely*?	Self-administered questionnaire	149 (2.0%)

For peer review only

Table 4c: Frequencies of self-reported sudden visual impairment across population studies

Study	Age Range	Date	Population	N	Question	How	Frequencies
Atherosclerosis Risk in Communities (ARIC) Cohort ***Toole et al (1996)*** [2]	45-64	1987-1989	Four centres in US adults residents	12,205	Have you ever had sudden "visual impairment"?	Administered by researcher – questions read aloud	313 (2.6%)
Reasons for geographic and racial differences in Stroke (REGARDS) ***Howard et al (2006)*** [4]	> 45 (mean 65)	2003	Community dwelling individuals in US. Those with stroke and TIA excluded	18462	Have you ever had sudden painless loss of vision in one or both eyes?	QVSFS questionnaire – telephone and in person	698 (3.8%)
Baltimore population study ***Mules et al (1971)*** [3]	>40	1954-1967	Population samples – two in US	9937	Loss of vision within 2 years prior to interview	Interview by trained interviewer	129 (2.0%)
Survey of Elderly Persons Living in Retirement Facilities ***Wilkinson et al (1979)*** [5]	>60	Published 1979	Elderly living in retirement facilities in 8 cities in the US	7281	During last **12 months** experienced sudden loss of eyesight *which subsequently cleared up completely*?	Self-administered questionnaire	149 (2.0%)

For peer review only

(1) Hart CL, Hole DJ, Smith GD. The relation between questions indicating transient ischaemic attack and stroke in 20 years of follow up in men and women in the Renfrew/Paisley Study. J Epidemiol Community Health 2001; 55(9):653-656.

(2) Toole JF, Lefkowitz DS, Chambless LE, Wijnberg L, Paton CC, Heiss G. Self-reported transient ischemic attack and stroke symptoms: methods and baseline prevalence. The ARIC Study, 1987-1989. Am J Epidemiol 1996; 144(9):849-856.

(3) Mules J, Henderson M, Kuller L. A population survey of symptoms suggestive of transient ischemic attacks. Stroke 1971; 2(2):114-127.

(4) Howard VJ, McClure LA, Meschia JF, Pulley L, Orr SC, Friday GH. High prevalence of stroke symptoms among persons without a diagnosis of stroke or transient ischemic attack in a general population: the REasons for Geographic And Racial Differences in Stroke (REGARDS) study. Arch Intern Med 2006; 166(18):1952-1958.

(5) Wilkinson WE, Heyman A, Burch JG, Ostfeld A, Labarthe DR, Leviton A et al. Use of a self-administered questionnaire for detection of transient cerebral ischemic attack

For peer review only

Table 2: Distribution and unadjusted and adjusted odds of transient neurological symptoms in those aged 65 or over in the Cognitive Function and Aging Study (CFAS)

(Data are count (%))

	Loss of Speech			Weakness			Loss of Sight			At least one symptom		
	**N**	**%***	**OR † (95%CI)**	**N**	**%***	**OR† (95%CI)**	**N**	**%***	**OR† (95%CI)**	**N**	**%***	**OR† (95%CI)**
**Gender**												
Male	119	2.5	1.0	257	5.5	1.0	347	7.6	1.0	598	12.8	1.0
Female	152	2.2	0.9(0.7-1.1)	339	4.8	0.9(0.7-1.0)	525	7.6	0.9(0.9-1.1)	858	12.0	0.9(0.7-1.1)
			0.9 (0.7-1.2)			0.9(0.7-1.0)			1.0(0.9-1.2)			1.0 (0.9-1.1)
**Age**												
65-74	137	2.3	1.0	302	5.1	1.0	480	8.1	1.0	768	12.9	1.0
75-84	104	2.3	1.0(0.8-1.3)	229	5.0	0.9(0.8-1.2)	327	7.2	0.9(0.7-1.0)	553	12.0	0.9(0.8-1.0)
			0.7(0.7-1.2)			0.9(0.7-1.1)			0.9(0.7-1.1)			0.9 (0.8-1.0)
85+	30	2.5	1.1(0.7-1.6)	65	5.4	1.1(0.8-1.4)	65	5.4	0.6(0.5-.08)	134	10.4	0.8(0.6-0.9)

For peer review only

			0.8(0.5-1.3)			0.9(0.6-1.2)			0.6(0.5-0.8)			0.7 (0.6-0.9)
**Social Class**												
Non-manual	96	5.8	1.0	63	11.0	1.0	295	17.9	1.0	497	10.2	1.0
Manual	164	7.2	1.3(.9-1.6)	390	17.8	1.6(1.3-1.9)	556	25.7	1.4(1.2-1.7)	920	14.1	1.4(1.3-1.6)
			1.1 (0.9-1.5)			1.4 (1.2-1.7)			1.4(1.2-1.6)			1.4 (1.2-1.6)
**Comorbidity**												
Angina (n=1538)	64	4.3	2.2(1.7-2.9)	176	11.7	3.1(2.6-3.8)	160	10.6	1.5(1.3-1.9)	312	20.7	2.1(1.8-2.4)
			1.5(1.0-2.2)			2.5(2.0-3.2)			1.3(1.0-1.6)			1.7(1.4-1.9)
Diabetes (n=672)	28	4.0	1.8(1.2-2.8)	40	6.1	1.2(.9-1.7)	66	10.1	1.4(1.0-1.8)	102	15.2	1.3(1.0-1.6)
			1.4(0.9-2.2)			1.0(0.7-1.4)			1.3(0.9-1.7)			1.1(0.9-1.4)
Heart Attack (n=1139)	52	4.7	2.3(1.7-3.2)	131	10.5	2.5(2.0-3.1)	119	11.5	1.7(1.4-2.1)	233	12.4	1.9(1.7-3.2)
			1.8(1.2-2.7)			1.4(1.2-1.9)			1.4(1.1-1.8)			1.4(1.2-1.8)

* Weighted percentages

†Adjusted for age, sex, social class, cognition and cardiovascular comorbidities

‡ Reference non-manual (I,II, IIIa) compared to manual social class (IIIb,IV,V)

For peer review only

156 participants had missing data on the speech questionnaire, 155 on the weakness and 154 on the sight question

For peer review only

For peer review only

Published by Oxford University Press on behalf of the International Epidemiological Association International Journal of Epidemiology 2006;35:1140–1145
© The Author 2006; all rights reserved. Advance Access publication 15 September 2006 doi:10.1093/ije/dyl199

COHORT PROFILE

Cohort Profile: The Medical Research Council Cognitive Function and Ageing Study (CFAS)†

Carol Brayne,[1]* Cherie McCracken[2] and Fiona E Matthews[3]

Origin of the study

Global ageing is a recent phenomenon. Its potential impact on social and economic aspects of more affluent countries highlighted ageing as a sufficiently important issue towards which to direct resources. Discussion between the Department of Health, Medical Research Council, and experts from the scientific and medical communities resulted in the decision that brain changes, most particularly cognitive decline, dementia, and their relation to disability were key topics requiring investigation at the population level. This prompted a decision to invest in research into this area and a working group was convened, which included those with epidemiological and biostatistical expertise relevant to such investigation. Out of this working group a successful bid for the study now known as the MRC Cognitive Function and Ageing Study emerged.

Study design

The study is a six-centre multidisciplinary multiphased longitudinal design (see map, Figure 1). There are five identical sites and one with a different sampling and interview structure. This centre (Liverpool) was already funded at the time of the discussions noted above and thus started earlier than the other five centres.[1] The other five centres (Cambridgeshire, Gwynedd, Newcastle, Nottingham, and Oxford) were able to follow a standardized design and are referred to as the five identical sites. Their basic structure was a two-phase design with a screening interview followed by an assessment interview shortly afterwards, with a repeat at 2 years. The fieldwork began in 1991.[2] There are many additional features, which are more fully described on the website (see below).

The aims of the study

The aims of the study have evolved over its existence and cover a wide range including descriptive epidemiology, neuropathology, policy, molecular epidemiology, and ethics.

The main descriptive epidemiological aims include (i) the estimation of the prevalence and incidence of cognitive decline and dementia, and geographical variation in those rates; (ii) the determination of the natural history of dementia, in particular the rate of progression of cognitive decline including the distribution of the interval between the identification of cognitive impairment and death, and (iii) the identification of factors associated with differing rates of cognitive decline and with the risk of dementia.

The principal neuropathological aim was to determine the contribution of different underlying pathologies to the rates of dementia and the geographic variation in these rates and to the burden of disability. Additional aims included to: (i) determine the prevalence and severity of pathological lesions in the brain of an unselected cohort of older people with and without cognitive impairment; (ii) determine the frequency of specific pathological diagnoses in people with cognitive impairment, and (iii) correlate severity of specific pathologies with patterns of cognition, function, and behaviour in life independently of clinical and pathological diagnostic categories.

The core aim related to policy was the evaluation of the degree of disability associated with cognitive decline and impairment, and the service needs this disability generates. These needs were to be compared with the needs generated by physical impairment. The study also sought to form the basis for longer-term studies of trends over time and by birth cohort of the prevalence and incidence of cognitive decline. In addition to these aims the breadth of the data collected has allowed the study to incorporate the investigation of expectation of life in various states of health, depression, and depressive symptomatology in the older population.

The DNA resource has been incorporated in a later phase of the study. The main molecular epidemiological aim has been to support genetics studies that have sought genes associated with all dementia, Alzheimer's disease, mixed and vascular dementia, cognitive impairment and decline.

A later aim of the study was to explore the ethical and legal aspects of brain donation within a population-based sample given changing perception surrounding organ donation.

The study also aimed to act as a core resource and provide a framework to support specific sub-studies in lone or joint centres. The Resource Implication Study[3–9] utilized this framework to achieve the core policy aim (see above). Other

[1] Department of Public Health and Primary Care, Institute of Public Health, Cambridge University, Forvie Site, Robinson Way, Cambridge CB2 2SR, UK.

[2] Department of Psychiatry, University of Liverpool University, Clinical Dept Duncan Building Liverpool L69 3BX, UK.

[3] MRC Biostatistics Unit, Institute of Public Health, Forvie Site, Robinson Way, Cambridge CB2 2SR, UK.

* Corresponding author. E-mail: Carol.Brayne@medschl.cam.ac.uk

† A full list of the study investigators and contributors is available on the website.

Downloaded from http://ije.oxfordjournals.org/ at Cambridge University Library on March 12, 2013

For peer review only

For peer review only - http://bmjopen.bmj.com/site/about/guidelines.xhtml

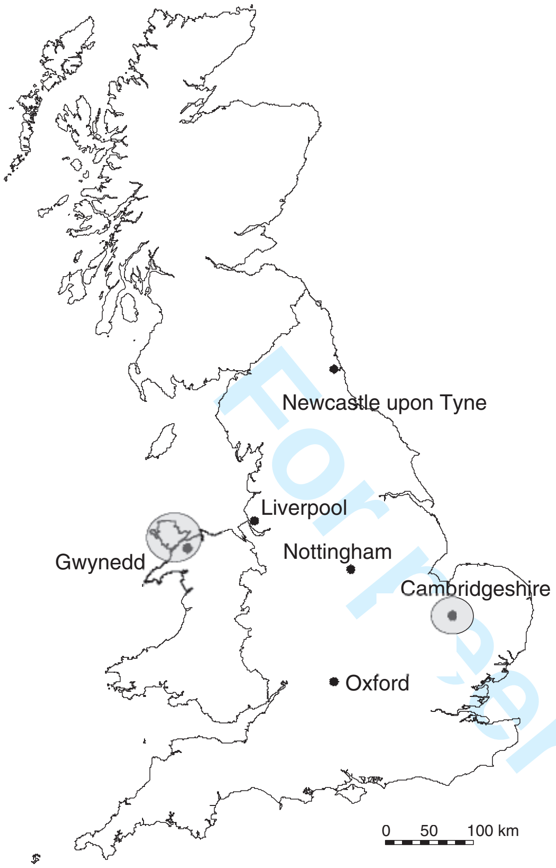

Figure 1 Map of Great Britain showing CFAS centres

sub-studies include the study of Healthy Ageing[10–15] and the Network Study. This framework also extends to wider collaborations with CFAS centres contributing to European wide initiatives such as EURODEM[16] and EURODEP.[17]

What does it cover?

Because the main study is focused on cognition and dementia it has collected the necessary dimensions of physical and mental health to arrive at a study diagnosis of dementia. It now has four major themes: (i) dementia (covering all aspects including cognition), (ii) depression, (iii) disability and healthy life expectancy, and (iv) health policy and health. In addition it has particular strengths in that it is one of the very few truly population based programmes with a donation programme—individuals in the study have indicated whether they wish to contribute to brain research through the donation of their brain after death (declaration of intention to donate).

Who is in the sample?

The first aim of the study was to estimate age-specific rates of prevalence of cognitive impairment and dementia among those aged 65 and over. The population is thus all those aged 65 years and over on the index date for centre (1990, 1991), living within a specified geographical location. Background information on the demographics of the populations sampled was collected from the Office of Population Census and Surveys (OPCS), 1990–91 census now Office of National Statistics (ONS), to relate to regional and national data.

Family Health Service Authority (FHSA) lists were used as the sampling frame. The frame would be incomplete if eligible members of the population were not registered with a GP. However individuals in long-stay hospitals remain registered with their GP 2 years after institutionalization so sampling from FHSA lists ensured their inclusion. Each centre looked into the practices of long-stay hospitals in their area to confirm this. The FHSA list of individuals was used for sampling on a geographical basis. Each centre defined this area, and the study population was drawn from all those who were resident within it. Problems of inaccuracy, patients who died or moved away but were still on the FHSA list, were resolved by asking GP surgeries to check the lists. On this basis, a sample of sufficient size to yield 2500 interviews of individuals aged 65 years and over, stratified by age (equal numbers aged 65–74 and 75 plus) was chosen from the FHSA lists for each selected area (in Liverpool this was 5000 interviews stratified by sex and 5 year age band). The population is flagged at ONS for mortality and the database is updated continuously.

The follow-up has been determined by funding and the design of associated bolt-on studies. The main follow-up waves for the identical sites are captured in the audit trail shown in Figure 2, which shows the numbers for the main screen, assessment, 1 year follow-up and 2 year rescreen, new selection for assessment and further 1 year follow-up, 6 year follow-up of the assessed (with venepuncture), 8 year follow-up of those with intentions to donate, and 10 year follow-up of the total sample. In addition to this the main associated studies are the Resource Implication Study (4 centres—Cambridgeshire, Newcastle, Nottingham, Oxford), which followed those who provided care to the physically and cognitively frail at baseline, the ESRC funded Healthy Ageing Project, which interviewed in detail those who were not selected into the Resource Implication Study in Nottingham and Cambridgeshire, the Network Study conducted in Gwynedd and Liverpool to examine individuals' social networks, an embedded case–control study at 2 year incidence stage (Cambridgeshire), and the ongoing brain donation programme in all centres. This programme, in combination with the bloods taken at year 6 form the major components of the Biological Resource of the study.

Who is not in the sample?

Comprehensive analyses of those who were lost to follow-up have been conducted for all stages of the study. At baseline 19% of potential respondents refused, 6% had died, and 1% had moved out of the area. Similar percentages were found for all waves of the study. Individuals who had moved or refused had higher mortality than responders.[18] CFAS has used this attrition for a detailed investigation of attrition effects in both short and longer time intervals.[18,19]

Downloaded from http://ije.oxfordjournals.org/ at Cambridge University Library on March 12, 2013

For peer review only

For peer review only - http://bmjopen.bmj.com/site/about/guidelines.xhtml

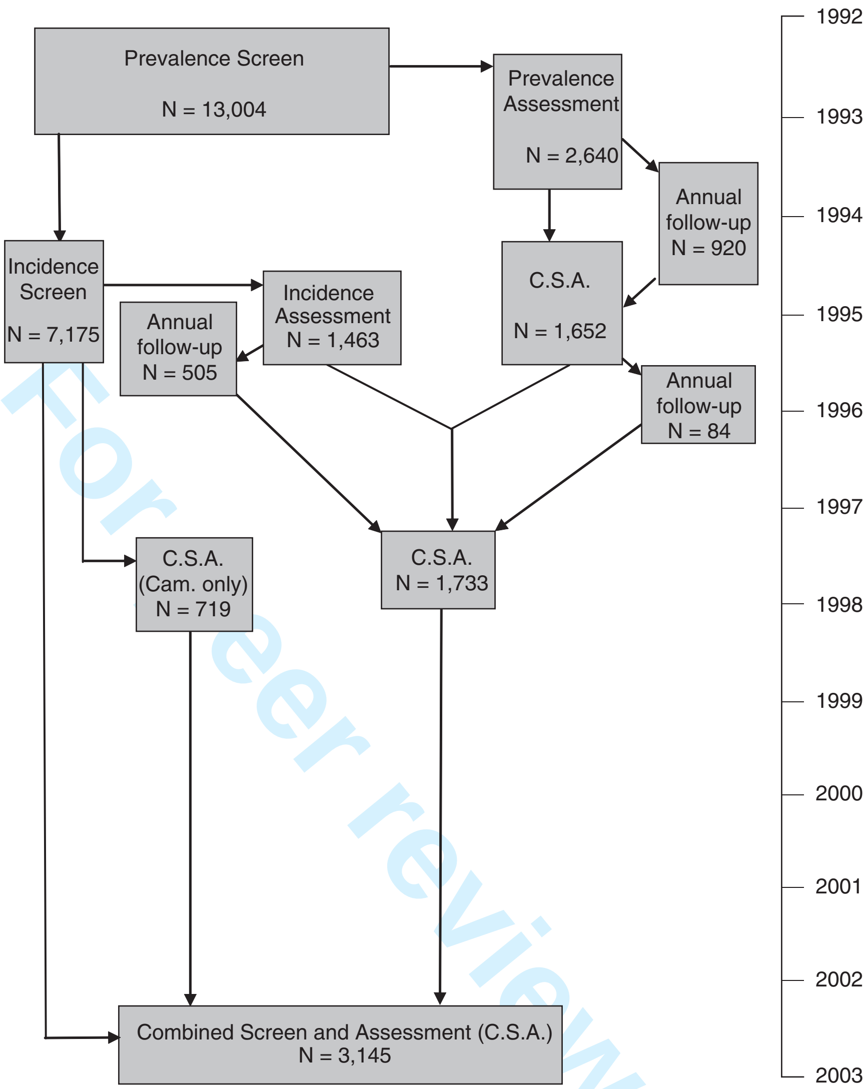

Figure 2 Time frame of CFAS interviews (identical centres)

How, when, and which interviews were conducted?

All interviews were conducted in the respondent's place of residence, using portable computers with customized software. If the interviewer felt that the respondent was frail and tiring, or becoming agitated, the short 'priority mode' set of questions could be invoked manually. Screening interviews were undertaken by lay interviewers, recruited for the purpose and trained by both the local and national coordinator. Reliability checks were made by both the local and the national coordinator. Proxy screening interviews were conducted where an interview was not possible with the named participant, owing to, for example, extreme confusion or frailty. If after four attempts to contact, an interview was not arranged, the approach was abandoned. The screening of the entire sample took 2 years to complete.

The assessment (designed to be conducted 1 month after the screening interview) and annual (designed to be conducted 1 year after the screening interview) interviews were also undertaken using customized portable computers. These interviews were carried out by interviewers from professions allied to medicine, who had not undertaken the screening interview, also recruited and trained for the purpose. Interviewers did not know the outcome of the first interview. The interviews lasted from 45 to 90 min, again with a 'priority mode' route. The annual interview consisted of a combined screen and assessment, where information on changes since last interview was recorded. At the assessment and biannual follow-up interview permission was sought to approach and interview a relative or carer to ask for an objective account of the respondent's health and abilities.

Six years after the initial screening interview, all respondents in the assessed groups were interviewed using the combined screen and assessment interview and at the end of that interview signed permission was requested to take a sample of blood or saliva. Permission was also sought to access GP and hospital notes. At 8 years only those who had indicated an

Downloaded from http://ije.oxfordjournals.org/ at Cambridge University Library on March 12, 2013

For peer review only

For peer review only - http://bmjopen.bmj.com/site/about/guidelines.xhtml

intention to donate brain tissue were re-interviewed with the combined screen and assessment interview.

At 10 years all survivors from the responding group of the complete study were recontacted for interview and if they agreed were interviewed using the combined screen and assessment interview.

What is collected at different interviews?

The screening interview contains questions on residence, marital status, education and occupation, living circumstances, contact with friends and family, health and social care contact, self-reported physical health, instrumental activities of daily living and activities of daily living, cognitive measures (Mini Mental State Examination with augmentation), and medication.

The assessment interview is mainly the Geriatric Mental State Examination (GMS) adapted for CFAS.[20] This is a structured psychiatric interview, which collects sufficient information for algorithmic 'diagnosis' in the major psychiatric disorders of old age (dementia, depression, anxiety, and psychosis). This has been validated against clinical diagnosis and the instrument has been widely used in Europe and now forms part of the 10/66 international instrument. This interview has been augmented with questions from the CAMDEX (Cambridge Examination for Mental Disorders in the Elderly) including CAMCOG,[21] the longer neuropsychological assessment. The relative or carer interview is mainly the History and Aetiological Schedule, the informant interview that accompanies the GMS.

The combined screen and assessment interview merges the two interviews but compresses some aspects of data collection. Complete versions of all the interviews including the interview questions and responses are available on the website.

The neuropathological assessment follows the standardized protocol of the Consortium to Establish a Registry for Alzheimer's Disease (CERAD)[22], with the exception that the neuropathologist is blind to the interview data. This covers in a semiquantitative form the main areas required for the assessment of neurodegenerative and cerebrovascular disorders. The forms are available on the website. The main genetic analyses have been on apoE and ACE.

Data collection in Liverpool was broadly similar to the other five sites except that the screening interview consisted of the GMS plus some of the screening interview questions listed above.

What has the study found?

The study has reported on prevalence and incidence of dementia, and lack of variation in these across the five identical sites.[23–25] It has provided profiles of cognition for MMSE, extended MMSE, and CAMCOG, weighted back to the population.[26,27] It has reported on risk for incident dementia including apoE and ACE.[28–30] It has examined the relationship of cognition to mortality.[18,31] It has reported on the mixed neuropathology found in the brains of the oldest old.[32] In addition, the study has reported on a variety of impairments to healthy life in old age and their population burden.[33–38] The Resource Implication Study has provided data for examination of carer burden and the costs of care for physically and cognitively frail.[3–9,39] The data have been used for projection forward, for these vulnerable groups and also for the costs of long-term care. Liverpool has published on the prevalence of dementia, depression, and neurosis, together with incidence of dementia and schizophrenia.[40–43]

What are the main strengths?

The study is multisite and multidisciplinary. The population is truly representative with high response rates at each stage over diverse sites. Where there is no heterogeneity across sites the study is sufficiently large to provide indicative values for national estimates. The broad scope of measures has allowed the study to contribute to ageing research across a wide range of topics. There are repeat measures on cognition and function, which allows examination of trajectories. There are only two other population-based studies with brain donation in Europe.[44,45] The study weighted the sample towards the over 75 age group at baseline, which has provided more robust data for the oldest old.

What are the main weaknesses?

It would be desirable to have higher response and lower drop out between waves, but analysis can adjust for loss between interviews. Blood taking and clinical assessment (including imaging) at baseline was not possible because of funding constraints, but venepuncture was included at year 6. The risk measures are self-report, using the available validated measures of the era.

Can I get hold of data?

The study actively encourages collaboration, and there are established mechanisms for approaching us via the themes mentioned above (see What does it cover?). Information is available on the website and also through contact with theme leads.

Where can I find out more?

The study website, www.cfas.ac.uk, configures information under themes, documentation, publications, and data. There is also a list of study contacts.

Acknowledgements

We are grateful to our respondents, their families, and their primary care teams. The study has only been possible because of the dedication of a large number of individuals over the years who are listed on the website. The MRC CFA Study has been supported by major awards from the Medical Research Council and the Department of Health. Thanks are also due to the Biological Resource Advisory Group for overseeing this aspect of study and to Lu Gao for preparation of the manuscript.

Downloaded from http://ije.oxfordjournals.org/ at Cambridge University Library on March 12, 2013

For peer review only

References

1 Saunders PA, Copeland JR, Dewey ME, Larkin BA, Scott A. Alpha: the Liverpool MRC Study of the incidence of dementia and cognitive decline. *Neuroepidemiology* 1992;**11**(suppl. 1):44–47.

2 Chadwick C. The MRC Multicentre Study of Cognitive Function and Ageing: a EURODEM incidence study in progress. *Neuroepidemiology* 1992;**11**(suppl. 1):37–43.

3 Buck D, Gregson BA, Bamford CH *et al*. Psychological distress among informal supporters of frail older people at home and in institutions. The Resource Implications Study Group of the MRC Cognitive Function and Ageing Study. *Int J Geriatr Psychiatry* 1997;**12**:737–44.

4 McNamee P, Gregson BA, Wright K, Buck D, Bamford CH, Bond J. Estimation of a multiproduct cost function for physically frail older people. *Health Econ* 1998;**7**:701–10.

5 McNamee P, Gregson BA, Buck D, Bamford CH, Bond J, Wright K. Costs of formal care for frail older people in England: the resource implications study of the MRC cognitive function and ageing study (RIS MRC CFAS). *Soc Sci Med* 1999;**48**:331–41.

6 Bond J, Farrow G, Gregson BA *et al*. Informal caregiving for frail older people at home and in long-term care institutions: who are the key supporters? *Health Soc Care Community* 1999;**7**:434–44.

7 Melzer D, McWilliams B, Brayne C, Johnson T, Bond J. Profile of disability in elderly people: estimates from a longitudinal population study. *BMJ* 1999;**318**:1108–11.

8 Psychological morbidity among informal caregivers of older people: a 2-year follow-up study. The Resource Implications Study Group of the MRC study of cognitive function and ageing (RIS MRC CFAS). *Psychol Med* 2000;**30**:943–55.

9 McNamee P, Bond J, Buck D. Costs of dementia in England and Wales in the 21st century. *Br J Psychiatry* 2001;**179**:261–66.

10 Solomou W, Richards MPM, Huppert FA, Brayne C, Morgan K. Divorce, current marital status and well-being in an elderly population. *Int J Law, Policy and the Family* 1998;**12**:321–342.

11 Huppert FA, Solomou W, O'Connor S, Morgan K, Sussams P, Brayne C. Aging and lymphocyte subpopulations: whole-blood analysis of immune markers in a large population sample of healthy elderly individuals. *Exp Gerontol* 1998;**33**:593–600.

12 Morgan K, Armstrong GK, Huppert FA, Brayne C, Solomou W. Healthy ageing in urban and rural Britain: a comparison of exercise and diet. *Age Ageing* 2000;**29**:341–48.

13 Huppert FA, Pinto EM, Morgan K, Brayne C. Survival in a population sample is predicted by proportions of lymphocyte subsets. *Mech Ageing Dev* 2003;**124**:449–51.

14 Huppert FA, Pinto E, Morgan K, MRC CFAS, Brayne C. Immune measures which predict 9-year survival in an elderly population sample. *Basic Biology and Clinical Impact of Immunosenescence, Advances in Cell Aging and Gerontology* 2003;**13**:17–28.

15 Pinto EM, Huppert FA, Morgan K, Mrc C, Brayne C. Neutrophil counts, monocyte counts and cardiovascular disease in the elderly. *Exp Gerontol* 2004;**39**:615–19.

16 Rocca WA, Hofman A, Brayne C *et al*. The prevalence of vascular dementia in Europe: facts and fragments from 1980–1990 studies. EURODEM-Prevalence Research Group. *Ann Neurol* 1991;**30**:817–24.

17 Copeland JR, Beekman AT, Braam AW *et al*. Depression among older people in Europe: the EURODEP studies. *World Psychiatry* 2004;**3**:45–49.

18 Matthews FE, Chatfield M, Freeman C, McCracken C, Brayne C. Attrition and bias in the MRC cognitive function and ageing study: an epidemiological investigation. *BMC Public Health* 2004;**4**:12.

19 Matthews FE, Chatfield M, Brayne C. An investigation of whether factors associated with short-term attrition change or persist over ten years: data from the Medical Research Council Cognitive Function and Ageing Study (MRC CFAS). *BMC Public Health* 2006;**6**:185.

20 Copeland JR, Dewey ME, Griffiths-Jones HM. A computerized psychiatric diagnostic system and case nomenclature for elderly subjects: GMS and AGECAT. *Psychol Med* 1986;**16**:89–99.

21 Huppert FA, Brayne C, Gill C, Paykel ES, Beardsall L. CAMCOG–a concise neuropsychological test to assist dementia diagnosis: socio-demographic determinants in an elderly population sample. *Br J Clin Psychol* 1995;**34**:529–41.

22 Mirra SS, Heyman A, McKeel D *et al*. The Consortium to Establish a Registry for Alzheimer's Disease (CERAD). Part II. Standardization of the neuropathologic assessment of Alzheimer's disease. *Neurology* 1991;**41**:479–86.

23 Cognitive function and dementia in six areas of England and Wales: the distribution of MMSE and prevalence of GMS organicity level in the MRC CFA Study. The Medical Research Council Cognitive Function and Ageing Study (MRC CFAS). *Psychol Med* 1998;**28**:319–35.

24 Matthews F, Brayne C. The incidence of dementia in England and Wales: findings from the five identical sites of the MRC CFA Study. *PLoS Med* 2005;**2**:e193.

25 Matthews FE, Dening T. Prevalence of dementia in institutional care. *Lancet* 2002;**360**:225–26.

26 Williams JG, Huppert FA, Matthews FE, Nickson J. Performance and normative values of a concise neuropsychological test (CAMCOG) in an elderly population sample. *Int J Geriatr Psychiatry* 2003;**18**:631–44.

27 Huppert FA, Cabelli ST, Matthews FE. Brief cognitive assessment in a UK population sample—distributional properties and the relationship between the MMSE and an extended mental state examination. *BMC Geriatr* 2005;**5**:7.

28 Yip AG, Brayne C, Matthews FE. Risk factors for incident dementia in England and Wales: The Medical Research Council Cognitive Function and Ageing Study. A population-based nested case-control study. *Age Ageing* 2006;**35**:154–60.

29 Yip AG, Brayne C, Easton D, Rubinsztein DC. An investigation of ACE as a risk factor for dementia and cognitive decline in the general population. *J Med Genet* 2002;**39**:403–06.

30 Yip AG, Brayne C, Easton D, Rubinsztein DC. Apolipoprotein E4 is only a weak predictor of dementia and cognitive decline in the general population. *J Med Genet* 2002;**39**:639–43.

31 Neale R, Brayne C, Johnson AL. Cognition and survival: an exploration in a large multicentre study of the population aged 65 years and over. *Int J Epidemiol* 2001;**30**:1383–88.

32 Pathological correlates of late-onset dementia in a multicentre, community-based population in England and Wales. Neuropathology Group of the Medical Research Council Cognitive Function and Ageing Study (MRC CFAS). *Lancet* 2001;**357**:169–75.

33 Brayne C, Gao L, Matthews F. Challenges in the epidemiological investigation of the relationships between physical activity, obesity, diabetes, dementia and depression. *Neurobiol Aging* 2005;**26**(suppl. 1):6–10.

34 Jagger C, Matthews F. Gender differences in life expectancy free of impairment at older ages. *J Women Aging* 2002;**14**:85–97.

35 McGee MA, Johnson AL, Kay DW. The description of activities of daily living in five centres in England and Wales. Medical Research Council Cognitive Function and Ageing Study. *Age Ageing* 1998;**27**:605–13.

36 Melzer D, McWilliams B, Brayne C, Johnson T, Bond J. Socioeconomic status and the expectation of disability in old age: estimates for England. *J Epidemiol Commun Health* 2000;**54**:286–92.

37 Parker CJ, Morgan K, Dewey ME. Physical illness and disability among elderly people in England and Wales: the Medical Research Council Cognitive Function and Ageing Study. The Analysis Group. *J Epidemiol Commun Health* 1997;**51**:494–501.

38 Spiers NA, Matthews RJ, Jagger C *et al*. Diseases and impairments as risk factors for onset of disability in the older population in England and

For peer review only

Downloaded from http://ije.oxfordjournals.org/ at Cambridge University Library on March 12, 2013

Wales: findings from the Medical Research Council Cognitive Function and Ageing Study. *J Gerontol A Biol Sci Med Sci* 2005;**60**:248–54.

39 Comas-Herrera A, Wittenberg R, Pickard L, Knapp M, MRC-CFAS. Cognitive impairment in older people: its implications for future demand for services and costs. *Mental Health Research Review* 2001;**8**:37–38.

40 Copeland JR, Chen R, Dewey ME *et al*. Community-based case-control study of depression in older people. Cases and sub-cases from the MRC-ALPHA Study. *Br J Psychiatry* 1999;**175**:340–47.

41 Copeland JR, McCracken CF, Dewey ME *et al*. Undifferentiated dementia, Alzheimer's disease and vascular dementia: age- and gender-related incidence in Liverpool. The MRC-ALPHA Study. *Br J Psychiatry* 1999;**175**:433–38.

42 Copeland JR, Dewey ME, Scott A *et al*. Schizophrenia and delusional disorder in older age: community prevalence, incidence, comorbidity, and outcome. *Schizophr Bull* 1998;**24**:153–61.

43 Saunders PA, Copeland JR, Dewey ME *et al*. The prevalence of dementia, depression and neurosis in later life: the Liverpool MRC-ALPHA Study. *Int J Epidemiol* 1993;**22**:838–47.

44 Polvikoski T, Sulkava R, Myllykangas L *et al*. Prevalence of Alzheimer's disease in very elderly people: a prospective neuropathological study. *Neurology* 2001;**56**:1690–96.

45 Xuereb JH, Brayne C, Dufouil C *et al*. Neuropathological findings in the very old. Results from the first 101 brains of a population-based longitudinal study of dementing disorders. *Ann N Y Acad Sci* 2000;**903**:490–96.

For peer review only

Downloaded from http://ije.oxfordjournals.org/ at Cambridge University Library on March 12, 2013

For peer review only - http://bmjopen.bmj.com/site/about/guidelines.xhtml

STROBE Statement—Checklist of items that should be included in reports of ***cohort studies***

- see below **

	Item No	Recommendation
**Title and abstract**	1	(*a*) Indicate the study's design with a commonly used term in the title or the abstract
		(*b*) Provide in the abstract an informative and balanced summary of what was done and what was found
**Introduction**		
Background/rationale	2	Explain the scientific background and rationale for the investigation being reported
Objectives	3	State specific objectives, including any prespecified hypotheses
**Methods**		
Study design	4	Present key elements of study design early in the paper
Setting	5	Describe the setting, locations, and relevant dates, including periods of recruitment, exposure, follow-up, and data collection
Participants	6	(*a*) Give the eligibility criteria, and the sources and methods of selection of participants. Describe methods of follow-up
		(*b*) For matched studies, give matching criteria and number of exposed and unexposed
Variables	7	Clearly define all outcomes, exposures, predictors, potential confounders, and effect modifiers. Give diagnostic criteria, if applicable
Data sources/ measurement	8*	For each variable of interest, give sources of data and details of methods of assessment (measurement). Describe comparability of assessment methods if there is more than one group
Bias	9	Describe any efforts to address potential sources of bias
Study size	10	Explain how the study size was arrived at
Quantitative variables	11	Explain how quantitative variables were handled in the analyses. If applicable, describe which groupings were chosen and why
Statistical methods	12	(*a*) Describe all statistical methods, including those used to control for confounding
		(*b*) Describe any methods used to examine subgroups and interactions
		(*c*) Explain how missing data were addressed
		(*d*) If applicable, explain how loss to follow-up was addressed
		(*e*) Describe any sensitivity analyses
**Results**		
Participants	13*	(a) Report numbers of individuals at each stage of study—eg numbers potentially eligible, examined for eligibility, confirmed eligible, included in the study, completing follow-up, and analysed
		(b) Give reasons for non-participation at each stage
		(c) Consider use of a flow diagram
Descriptive data	14*	(a) Give characteristics of study participants (eg demographic, clinical, social) and information on exposures and potential confounders
		(b) Indicate number of participants with missing data for each variable of interest
		(c) Summarise follow-up time (eg, average and total amount)
Outcome data	15*	Report numbers of outcome events or summary measures over time
Main results	16	(*a*) Give unadjusted estimates and, if applicable, confounder-adjusted estimates and their precision (eg, 95% confidence interval). Make clear which confounders were adjusted for and why they were included
		(*b*) Report category boundaries when continuous variables were categorized

For peer review only

		(*c*) If relevant, consider translating estimates of relative risk into absolute risk for a meaningful time period
Other analyses	17	Report other analyses done—eg analyses of subgroups and interactions, and sensitivity analyses
**Discussion**		
Key results	18	Summarise key results with reference to study objectives
Limitations	19	Discuss limitations of the study, taking into account sources of potential bias or imprecision. Discuss both direction and magnitude of any potential bias
Interpretation	20	Give a cautious overall interpretation of results considering objectives, limitations, multiplicity of analyses, results from similar studies, and other relevant evidence
Generalisability	21	Discuss the generalisability (external validity) of the study results
**Other information**		
Funding	22	Give the source of funding and the role of the funders for the present study and, if applicable, for the original study on which the present article is based

*Give information separately for exposed and unexposed groups.

Note: An Explanation and Elaboration article discusses each checklist item and gives methodological background and published examples of transparent reporting. The STROBE checklist is best used in conjunction with this article (freely available on the Web sites of PLoS Medicine at http://www.plosmedicine.org/, Annals of Internal Medicine at http://www.annals.org/, and Epidemiology at http://www.epidem.com/). Information on the STROBE Initiative is available at http://www.strobe-statement.org.

STROBE checklist

Item	Reported on page
1(a)	1
1(b)	2
2	3,4
3	4
4	4,5
5	4-7
6 (a)	4,5
7	6
8	5,6
9	6
10	Described elsewhere - see references 1 and 2
11	6
12 (a)	6,7
12 (b)	6,7
12 (c)	6,7
12 (d)	6,7 – described elsewhere see references 1 and 2
12 (e)	N/A
13 (a)	7
13 (b)	7
13(c)	Described elsewhere – see reference 1
14 (a)	7
14(b)	see tables
14 (c)	6

For peer review only - http://bmjopen.bmj.com/site/about/guidelines.xhtml
For peer review only

15 cohort study	N/A
16(a)	see additional table
16(b)	N/A
16 (c)	N/A
17	7/8
18	7/8
19	11
20	9/10
21	9/10
22	13

Reference 1:

Brayne C, McCracken C, Matthews FE. Cohort profile: the Medical Research Council Cognitive Function and Ageing Study (CFAS). *Int J Epidemiol* 2006; 35(5):1140-1145.

Matthews FE, Chatfield M, Freeman C, McCracken C, Brayne C, MRC CFAS (2004), "Attrition and bias in the MRC cognitive function and ageing study: an epidemiological investigation." *BMC Public Health* 4:12

For peer review only

Please wait...

If this message is not eventually replaced by the proper contents of the document, your PDF viewer may not be able to display this type of document.

You can upgrade to the latest version of Adobe Reader for Windows®, Mac, or Linux® by visiting http://www.adobe.com/go/reader_download.

For more assistance with Adobe Reader visit http://www.adobe.com/go/acrreader.

Windows is either a registered trademark or a trademark of Microsoft Corporation in the United States and/or other countries. Mac is a trademark of Apple Inc., registered in the United States and other countries. Linux is the registered trademark of Linus Torvalds in the U.S. and other countries.

Please wait...

If this message is not eventually replaced by the proper contents of the document, your PDF viewer may not be able to display this type of document.

You can upgrade to the latest version of Adobe Reader for Windows®, Mac, or Linux® by visiting http://www.adobe.com/go/reader_download.

For more assistance with Adobe Reader visit http://www.adobe.com/go/acrreader.

Windows is either a registered trademark or a trademark of Microsoft Corporation in the United States and/or other countries. Mac is a trademark of Apple Inc., registered in the United States and other countries. Linux is the registered trademark of Linus Torvalds in the U.S. and other countries.

Please wait...

If this message is not eventually replaced by the proper contents of the document, your PDF viewer may not be able to display this type of document.

You can upgrade to the latest version of Adobe Reader for Windows®, Mac, or Linux® by visiting http://www.adobe.com/go/reader_download.

For more assistance with Adobe Reader visit http://www.adobe.com/go/acrreader.

Windows is either a registered trademark or a trademark of Microsoft Corporation in the United States and/or other countries. Mac is a trademark of Apple Inc., registered in the United States and other countries. Linux is the registered trademark of Linus Torvalds in the U.S. and other countries.

Please wait...

If this message is not eventually replaced by the proper contents of the document, your PDF viewer may not be able to display this type of document.

You can upgrade to the latest version of Adobe Reader for Windows®, Mac, or Linux® by visiting http://www.adobe.com/go/reader_download.

For more assistance with Adobe Reader visit http://www.adobe.com/go/acrreader.

Windows is either a registered trademark or a trademark of Microsoft Corporation in the United States and/or other countries. Mac is a trademark of Apple Inc., registered in the United States and other countries. Linux is the registered trademark of Linus Torvalds in the U.S. and other countries.

Please wait...

If this message is not eventually replaced by the proper contents of the document, your PDF viewer may not be able to display this type of document.

You can upgrade to the latest version of Adobe Reader for Windows®, Mac, or Linux® by visiting http://www.adobe.com/go/reader_download.

For more assistance with Adobe Reader visit http://www.adobe.com/go/acrreader.

Windows is either a registered trademark or a trademark of Microsoft Corporation in the United States and/or other countries. Mac is a trademark of Apple Inc., registered in the United States and other countries. Linux is the registered trademark of Linus Torvalds in the U.S. and other countries.

Please wait...

If this message is not eventually replaced by the proper contents of the document, your PDF viewer may not be able to display this type of document.

You can upgrade to the latest version of Adobe Reader for Windows®, Mac, or Linux® by visiting http://www.adobe.com/products/acrobat/readstep2.html.

For more assistance with Adobe Reader visit http://www.adobe.com/support/products/acrreader.html.

Windows is either a registered trademark or a trademark of Microsoft Corporation in the United States and/or other countries. Mac is a trademark of Apple Inc., registered in the United States and other countries. Linux is the registered trademark of Linus Torvalds in the U.S. and other countries.

Transient neurological symptoms in the older population: Report of a prospective cohort study - the Medical Research Council Cognitive Function and Ageing (CFAS) Study

Journal:	*BMJ Open*
Manuscript ID:	bmjopen-2013-003195.R1
Article Type:	Research
Date Submitted by the Author:	11-Jun-2013
Complete List of Authors:	Mavaddat, Nahal; University of Cambridge, Department of Public Health and Primary Care<br>Savva, George; University of East Anglia, School of Nursing Sciences<br>Lasserson, Daniel; University of Oxford, Primary Care Health Sciences<br>giles, matthew; John Radcliffe Hospital, NIHR Biomedical Research Unit<br>Brayne, Carol; University of Cambridge, Institute of Public Health<br>Mant, Jonathan; University of Cambridge, General Practice and Primary Care Research Unit
**Primary Subject Heading**:	Neurology
Secondary Subject Heading:	Cardiovascular medicine, Epidemiology
Keywords:	Stroke < NEUROLOGY, EPIDEMIOLOGY, Stroke medicine < INTERNAL MEDICINE

SCHOLARONE™ Manuscripts

For peer review only

Transient neurological symptoms in the older population: Report of a prospective cohort study - the Medical Research Council Cognitive Function and Ageing Study (CFAS)

Nahal Mavaddat, George M Savva, Daniel Lasserson, Matthew Giles, Carol Brayne, Jonathan Mant

Department of Public Health and Primary Care, University of Cambridge, Strangeways Laboratory, Worts Causeway, Cambridge, UK CB1 8RN Nahal Mavaddat clinical lecturer Jonathan Mant professor

School of Nursing Sciences, University of East Anglia, Norwich Research Park, Norwich, UK, NR4 7TJ. George Savva senior lecturer

Department of Primary Care Health Sciences, University of Oxford, Oxford, U.K. New Radcliffe House, 2nd floor, Walton Street, Jericho, UK OX2 6NW Daniel Lasserson clinical lecturer

Stroke Prevention Research Unit, NIHR Biomedical Research Centre, Oxford University, Department of Clinical Neurology, John Radcliffe Hospital, Oxford, UK OX3 9DU Matthew Giles consultant physician

Department of Public Health and Primary Care, University of Cambridge, Institute of Public Health, Forvie Site, Robinson Way Causeway, Cambridge, UK CB2 0SR Carol Brayne professor

Correspondence to: Nahal Mavaddat nm212@medschl.cam.ac.uk

The Corresponding Author has the right to grant on behalf of all authors and does grant on behalf of all authors, an exclusive licence (or non exclusive for government employees) on a worldwide basis to the BMJ Publishing Group Ltd to permit this article (if accepted) to be published in BMJ editions and any other BMJPGL products and sublicences such use and exploit all subsidiary rights, as set out in our licence.

For peer review only

For peer review only

Article Summary

Article Focus:

- Prompt initiation of secondary prevention following TIA is associated with up to 80% reduction in risk of subsequent stroke.
- Many people presenting to specialist TIA clinics with transient neurological symptoms do not have TIA.
- The prevalence and incidence of transient neurological symptoms (as opposed to TIA) in older age groups is unknown.

Key Messages:

- In a large multi-centred community based study representative of the older population in the UK, we found a high prevalence and incidence of transient neurological symptoms, significantly greater than that of TIAs in hospital and population-based studies.

The high rates of transient neurological symptoms in the community in the older population should be considered when planning TIA services.

Strengths and Limitations of this study:

- Strengths - Previous studies of incidence and prevalence of transient neurological symptoms have mostly been conducted in populations not representative of older age groups where TIA is most common and few have been undertaken in UK populations.
- Limitations - The incidence of transient neurological symptoms in the study was determined in people without severe cognitive impairment, and may be an underestimate since cognitive impairment can be a manifestation of vascular disease.

For peer review only

The wording of questions to identify transient neurological symptoms may have picked up some people who had symptoms lasting more than 24 hours.

For peer review only

ABSTRACT

Objective: Transient ischaemic attack (TIA) is a recognised risk factor for stroke in the older population requiring timely assessment and treatment by a specialist. The need for such TIA services is driven by the epidemiology of transient neurological symptoms, which may not be caused by TIA. We report prevalence and incidence of transient neurological symptoms in a large UK cohort study of older people. **Design** Longitudinal cohort study **Setting and Participants:** The MRC Cognitive Function and Aging Study (CFAS) is a population representative study derived from random samples of people in their 65th year. Interview at baseline included questions about stroke and three transient neurological symptoms, repeated in a sub-sample after two years. Patients were flagged for mortality. **Main Outcome Measures** Prevalence and two year incidence of transient neurological symptoms. **Results** In 11,903 participants without a history of stroke, 271(2.3%) reported transient problems with speech, 872 (7.6%) with sight, and 596 (5.1%) weakness in a limb, with 1456 (12.7%) reporting at least one symptom. Of those re-interviewed (n=6748), 675 (9.8%) reported at least one symptom over two years. **Conclusions** Lifetime prevalence and incidence of transient neurological symptoms in people 65 and over is high and is substantially greater than the incidence of TIA in hospital and population-based studies. These high rates of transient neurological symptoms in the community in the older population should be considered when planning TIA services.

INTRODUCTION

For peer review only

Transient ischaemic attack (TIA) is an established and powerful risk factor for stroke. 8% of patients who have a TIA suffer a stroke within 7 days, many within 48 hours.[1;2] Immediate specialist assessment and treatment is associated with substantial reductions in this early risk of stroke, [3] and is recommended for people with suspected TIA. [4]

As awareness grows of the urgency of early management of TIA amongst both primary care practitioners and the general public, [5] more patients with symptoms suggestive of a TIA are likely to present to primary and secondary care services. Need for specialist services will be driven by incidence of symptoms that might represent TIA (i.e. transient neurological symptoms) rather than the epidemiology of TIA *per se*. Currently about 48,000 probable or definite TIAs (transient neurological symptoms lasting less than 24 hours of likely vascular aetiology), and 43,000 minor strokes (stroke events causing minimal or no neurological deficits) are managed as outpatients every year in England, but it is not clear to what extent this might be the tip of the iceberg. [6] Data are available worldwide on the prevalence and incidence of TIA. [7-11] The epidemiology in the community of transient neurological symptoms (i.e. neurological symptoms of sudden onset of which TIA is a subset), is however less well defined. Previous studies of incidence and prevalence of transient neurological symptoms, have mostly been conducted in populations not representative of older age groups where TIA is most common. [7;11-16] Very few have been undertaken in UK populations. While incidence of transient neurological symptoms will drive need for specialist TIA services, prevalence of transient neurological symptoms in the community is also important. TIA is associated with a long term increase in risk of stroke, [17;18] so there may be value in diagnosing 'old' events that have not presented to medical services in order to target

For peer review only

secondary prevention. Hence the need to know both the incidence and prevalence of transient neurological symptoms in the community.

This study reports prevalence and incidence of three common transient neurological symptoms (limb weakness, loss of speech and disturbance of vision) in a population based multi-centred cohort study in the UK (England and Wales) in those aged 65 and over, the Medical Research Council Cognitive Function and Ageing Study (CFAS). [19]

METHODS

The Medical Research Council CFAS is a population representative study of individuals aged 65 years and over. The study began in 1991 and was designed to determine the incidence of dementia in the older population. It has six centres across England and Wales chosen to represent the national variation of urban-rural mix, socio-economic deprivation and rates of chronic disease.[19] Five of these with identical study designs (Oxford, Nottingham, Cambridgeshire Gwynedd and Newcastle) are used in the present investigation. The sixth centre (Liverpool) used a different design and is therefore excluded. Random samples of people in their 65th year and above were obtained from Family Health Service Authority lists (agency responsible for maintaining registers of general practice populations at that time). The sample was stratified by age (65-74, and 75 years and over) and equal numbers were randomly selected from these groups with the aim of recruiting 2500 to each centre. All study centres obtained ethical approval from local research committees. (REC Ref: 05/MRE05/37). Full details of methodology are available elsewhere. [19]

Eligible participants (or their proxies where appropriate) provided informed consent. Trained interviewers undertook baseline interviews in the participants' homes including socio-demographic characteristics, cognitive function and disease history including previous

For peer review only

stroke, coronary heart disease and diabetes (full details at www.cfas.ac.uk). Based on baseline screening, the study sample was divided into two groups at baseline: people without cognitive impairment and a group consisting of those with cognitive impairment plus a stratified subsample of those without cognitive impairment. The first group underwent no further assessment at baseline and were 'rescreened' after two years. The second group underwent a further detailed cognitive assessment at baseline, but were not followed up for transient neurological symptoms. The estimates of prevalence of transient neurological symptoms are calculated for all people at baseline, while the incidence estimates use only the first group. All participants were flagged with the NHS Central Register. Deaths and underlying causes of death attributed to stroke (ICD codes 430-438) were notified to the study.

Prevalence of transient neurological symptoms

At baseline all participants were asked "Have you ever experienced sudden problems with:

(1) speech, which got better after a day?

(2) weakness in the arms or legs, which got better after a day?

(3) sight, which got better after a day?"

Social class was determined using the Registrar General's Occupational Classification. Cognitive status was determined using the Mini Mental State Examination (MMSE), [20] and the Automated Geriatric Examination for Computer Assisted Taxonomy (AGECAT). [21]

Incidence of transient neurological symptoms

All those in the 'rescreen' arm were asked at two year follow-up if they had experienced each transient neurological symptom in the past two years.

For peer review only

Analysis

All analyses were carried out using STATA version 11. Inverse probability weights were used throughout to ensure the sample was representative of the target population. Weights were estimated with logistic regression using presence in each phase of the study as an outcome and taking into account over-sampling of over-75s at baseline. Weights for incidence calculations adjust for attrition based on baseline characteristics and stratified selection into the assessment arm as appropriate. Baseline prevalence of each transient neurological symptom was calculated for age specific strata and as a weighted percentage to provide a population estimate of prevalence for people aged 65 and over. Associations with gender, age, social class (manual (IIIb, IV, V) and non-manual (I, II, IIIa)), and cardiovascular comorbidity were explored using logistic regression models adjusting for all other factors and cognitive function based on MMSE by score (less than 18, 18-21, 22-25 and 26-30).

Two year incidence of transient neurological symptoms was estimated using weighted percentages of those reporting any of the symptoms during follow-up. Weights were adjusted for refusals, drop-outs and for non-reassessment of people with cognitive impairment. Calculation of attrition weights for incident transient neurological symptoms excluded those who died between baseline and follow up. These estimates are therefore applicable to the population over 65 without severe cognitive impairment or stroke at baseline surviving at least two years.

RESULTS

The participation rate in the CFAS study was 80% (13,004/16,258). For the prevalence analysis, participants who had a stroke at baseline (963, 7.4%) or for whom baseline

For peer review only

information about stroke was missing (138, 1.1%) were excluded, leaving 11903 participants. 2,283 of the original participants were not allocated to the 'rescreen' arm at two years; 754 died, 1973 declined to participate and 145 were lost to follow-up, leaving 6,748 (76% response rate of potential participants) for the incidence analysis.

Table 1 shows the demographic features of participants in the CFAS studied in this analysis at baseline. 40% of participants were male and 60% of participants were female. 12.3% of all participants reported at least one transient neurological symptom. 2.3% reported transient loss of speech, 5.1% transient weakness and 7.6% transient loss of sight.

Table 2 shows prevalence data with weighted percentages and odds ratios (adjusted for age, sex, social class, cognition and cardiovascular morbidities) of reported transient neurological symptoms at baseline by demographic factors and comorbidities. 12.8% of males and 12.0% of females reported at least one of the three transient neurological symptoms of loss of speech, loss of sight, or weakness. There was no significant association of gender with reporting at least one transient neurological symptom, but significantly lower odds of reporting a transient neurological symptom in the over 85 age group. The odds of reporting any transient neurological symptom were higher in those in manual compared to non-manual social classes. The presence of cardiovascular morbidities of angina and heart attack were also significantly associated with the odds of having at least one of the transient neurological symptoms.

There was no difference between genders with regards to reporting individual transient neurological symptoms of loss of speech, loss of sight or weakness. However, there was a significantly lower odds of reporting transient visual loss in those over 85, and an increased odds of reporting transient symptoms of weakness and loss of sight in manual compared to

For peer review only

non-manual social classes. The presence of cardiovascular co-morbidities of angina and heart attack were associated with higher odds of all three symptoms.

Table 3 shows two year incidence of transient neurological symptoms in respondents attending the "rescreen". 9.8% of all participants reported at least one transient neurological symptom over the 2 years, with the highest incidence of reported transient neurological symptom being due to loss of sight followed by weakness, with loss of speech being the least frequently reported transient neurological symptom. The incidence of each of the transient neurological symptoms was highest in the 75 to 84 age group and lowest in the over 85 age group.

DISCUSSION

Our findings suggest that transient neurological symptoms are common in the older population in England and Wales, with at least 12% of people aged 65 and over having experienced a transient neurological symptom of the arm or leg, speech or vision better after a day, and approximately 5% experiencing at least one such symptom over the course of a year. The commonest of these was transient symptoms of vision, followed by limb weakness. Those over 85 reported lower rates of transient neurological symptoms, predominantly due to less frequent reporting of transient visual loss. Problems of memory and recall may have contributed to under-reporting, and higher mortality rates associated with neurological incidents to underestimation of rates of transient neurological symptoms in this age group. Chronic visual problems may have also potentially masked transient visual losses in the oldest-old population. Incidence of at least one transient neurological symptom in the CFAS population was approximately 2.6 times greater than that of confirmed TIA presenting to

For peer review only

medical services in the Oxford Vascular Study (OxVASC) in people aged over 85 (approximately 15 per 1000 per year), 8 times greater in those aged 75 to 84 (7 per 1000 per year), and approximately 15 times greater in those aged 65 to 74 (3 per 1000 population per year). [10]

The prevalence and incidence of transient neurological symptoms in the CFAS was somewhat higher than those found by questionnaires used in other studies internationally (mostly conducted in younger age groups), but comparable to those of Wilkinson et al. in an over 60 age group in a US based study. [7;12-14;16] Table 4 (online) shows the prevalence of sudden onset of neurological symptoms of weakness in a limb or of loss of speech or sight in previous population and community studies. [7;12-14;16] Questions used to elicit transient neurological symptoms in the CFAS are likely to have captured greater numbers of transient neurological events in the population compared to those seeking more specific vascular symptoms, or to the few studies defining more precisely the onset, offset and timing of the event. [7;12-14] While some respondents will have been describing true transient ischaemic events, it is likely that many did not have a true TIA. Validated measures to determine the presence of previous transient ischaemic attack are limited, and most studies of transient neurological symptoms in questionnaires overestimate true transient ischaemic attacks. [13;14;22] Wilkinson et al. (1979) suggest that around 10% of transient symptoms reported in a questionnaire are subsequently diagnosed as TIA after neurologist assessment.[14] Self-reported transient neurological symptoms in other community based studies have low positive predictive value, with higher values only in studies in outpatient populations (over 70%). [13;14;22-24] This suggests that the use of screening to identify possible past transient ischaemic attacks may generate unnecessary extra strain on primary care and secondary TIA services with limited benefit.

For peer review only

Patients presenting with transient neurological symptoms present considerable diagnostic dilemmas for primary care practitioners reflected in low rates of TIA diagnosis confirmation in patients referred to UK TIA clinics (over 50% being for non-TIA causes). [6;25;26] Many symptoms of TIA are non-specific and occur in non-vascular syndromes. Dizziness, ophthalmological problems, migraine, epilepsy, nerve entrapment or psychological states are the commonest non-TIA diagnoses. [25-29] Often, however, no diagnosis can be determined. [25;26] Scales such as the ABCD2 [2] help determine the urgency of TIA referral, but do not distinguish TIA from non-TIA symptoms. Wilkinson et al. reported transient loss of speech and loss of sight to be more reliable than limb weakness for neurologist diagnosis of TIA, [14] while Hart et al. found the strongest and most consistent relationship with subsequent stroke to be loss of power in an arm.[7] Other studies suggest that the presence of certain symptoms such as headache, dizziness, loss of consciousness, memory loss, blurred vision, generalised weakness, pain in limbs and seizures make the diagnosis of TIA less likely. [14;29;30]

Study limitations

The incidence of transient neurological symptoms was determined in people without severe cognitive impairment, and may be an underestimate since cognitive impairment can be a manifestation of vascular disease. [31;32] Only three transient neurological symptoms were reported in this study. Some symptoms of TIA (e.g. posterior circulation or pure sensory symptoms) were not sought. The wording of questions to identify transient neurological symptoms may have picked up some people who had symptoms lasting more than 24 hours. Such patients are nevertheless likely to draw on TIA services for further assessment. In addition, it was not possible to determine whether different transient neurological symptoms in a participant occurred simultaneously or separately in time. The problem of recall bias in

For peer review only

these self-reports of transient symptoms needs to be considered. Questions were designed to be easily comprehensible to the older population. However, the need to consider multiple criteria when responding to a question e.g. “sudden onset” and “less than a day”, may have led to difficulties in interpretation of the transient neurological symptoms questions for some participants. Finally, baseline and two year follow-up were carried out in the 1990s and changes in rates of vascular events may have occurred since then. While the age-specific incidence of stroke appears to be declining, it is not clear that the same is true of TIA. [10;33;34] As well, this study is more recent than many studies estimating rates of transient neurological symptoms in the population, many of which have been carried out during the 1970’s or ‘80’s. [12-14]

Conclusion

In a large multi-centred community based study highly representative of the older population in the UK and conducted in the age group where TIAs are most common, we found a high prevalence and incidence of transient neurological symptoms. The incidence of such symptoms in the community is significantly greater than that of TIAs. This highlights the need for adequate provision of TIA services and the potential importance of the development of valid diagnostic tools to assist the general practitioner and hospital doctor in better triage of people presenting with transient neurological symptoms.

ACKNOWLEDGEMENTS

We thank the MRC CFAS study group for data collection and management. We are also grateful to all respondents, their families and their primary care teams from across the country for their participation in MRC CFAS.

For peer review only

COMPETING INTERESTS

"All authors have completed the ICMJE uniform disclosure form at www.icmje.org/coi_disclosure.pdf and declare: no support from any organisation for the submitted work; no financial relationships with any organisations that might have an interest in the submitted work in the previous three years; no other relationships or activities that could appear to have influenced the submitted work."

FUNDING

The MRC CFAS Study has had funding by major awards from the Medical Research Council (MRC/G9901400) and Department of Health. NM was funded by a National Institute for Health Research (NIHR) Walport Clinical Lectureship in Primary Care Research. None of the sponsors influenced the design or conduct of the study or the analysis or interpretation of the findings. All researchers acted independently from funders in the design and carrying out of this study.

AUTHORS CONTRIBUTIONS

CB is Principal Investigator of the MRC CFAS Study. NM and GS contributed to analysis of data. All authors contributed to study design and intellectual input. All authors, external and internal, had full access to all of the data (including statistical reports and tables) and can take responsibility for the integrity of the data and accuracy of the data analysis.

DATA SHARING

Data sharing through application.

For peer review only

Reference List

(1) Coull AJ, Lovett JK, Rothwell PM. Population based study of early risk of stroke after transient ischaemic attack or minor stroke: implications for public education and organisation of services. *BMJ* 2004; 328(7435):326.

(2) Rothwell PM, Giles MF, Flossmann E, et al. A simple score (ABCD) to identify individuals at high early risk of stroke after transient ischaemic attack. *Lancet* 2005; 366(9479):29-36.

(3) Rothwell PM, Giles MF, Chandratheva A, et al. Effect of urgent treatment of transient ischaemic attack and minor stroke on early recurrent stroke (EXPRESS study): a prospective population-based sequential comparison. *Lancet* 2007; 370(9596):1432-1442.

(4) Department of Health. Department of Health. National Stroke Strategy. 2007.

(5) Department of Health. Stroke: Act F.A.S.T. awareness campaign. 2009. Department of Health.

(6) Giles MF, Rothwell PM. Substantial underestimation of the need for outpatient services for TIA and minor stroke. *Age Ageing* 2007; 36(6):676-680.

(7) Hart CL, Hole DJ, Smith GD. The relation between questions indicating transient ischaemic attack and stroke in 20 years of follow up in men and women in the Renfrew/Paisley Study. *J Epidemiol Community Health* 2001; 55(9):653-656.

(8) Mant J, Wade DT, Winner S. Health care needs assessment: stroke. In: Stevens A, Raftery J, Mant J, Simpson S, editors. Health care needs assessment: the epidemiologically based needs assessment reviews. 2nd ed. Oxford: Radcliffe Medical Press; 2004. 141-244.

(9) Orlandi G, Gelli A, Fanucchi S, et al. Prevalence of stroke and transient ischaemic attack in the elderly population of an Italian rural community. *Eur J Epidemiol* 2003; 18(9):879-882.

(10) Rothwell PM, Coull AJ, Silver LE, et al. Population-based study of event-rate, incidence, case fatality, and mortality for all acute vascular events in all arterial territories (Oxford Vascular Study). *Lancet* 2005; 366(9499):1773-1783.

(11) Bos MJ, van Rijn MJ, Witteman JC, et al. Incidence and prognosis of transient neurological attacks. *JAMA* 2007; 298(24):2877-2885.

(12) Mules J, Henderson M, Kuller L. A population survey of symptoms suggestive of transient ischemic attacks. *Stroke* 1971; 2(2):114-127.

(13) Toole JF, Lefkowitz DS, Chambless LE, et al. Self-reported transient ischemic attack and stroke symptoms: methods and baseline prevalence. The ARIC Study, 1987-1989. *Am J Epidemiol* 1996; 144(9):849-856.

For peer review only

(14) Wilkinson WE, Heyman A, Burch JG, et al. Use of a self-administered questionnaire for detection of transient cerebral ischemic attacks: I. Survey of elderly persons living in retirement facilities. *Ann Neurol* 1979; 6(1):40-46.

(15) Bots ML, van der Wilk EC, Koudstaal PJ, et al. Transient neurological attacks in the general population. Prevalence, risk factors, and clinical relevance. *Stroke* 1997; 28(4):768-773.

(16) Howard VJ, McClure LA, Meschia JF, et al. High prevalence of stroke symptoms among persons without a diagnosis of stroke or transient ischemic attack in a general population: the REasons for Geographic And Racial Differences in Stroke (REGARDS) study. *Arch Intern Med* 2006; 166(18):1952-1958.

(17) van Wijk I, Kappelle LJ, van GJ, et al. Long-term survival and vascular event risk after transient ischaemic attack or minor ischaemic stroke: a cohort study. *Lancet* 2005; 365(9477):2098-2104.

(18) Clark TG, Murphy MF, Rothwell PM. Long term risks of stroke, myocardial infarction, and vascular death in "low risk" patients with a non-recent transient ischaemic attack. *J Neurol Neurosurg Psychiatry* 2003; 74(5):577-580.

(19) Brayne C, McCracken C, Matthews FE. Cohort profile: the Medical Research Council Cognitive Function and Ageing Study (CFAS). *Int J Epidemiol* 2006; 35(5):1140-1145.

(20) Folstein MF, Folstein SE, McHugh PR. "Mini-mental state". A practical method for grading the cognitive state of patients for the clinician. *J Psychiatr Res* 1975; 12(3):189-198.

(21) Copeland JR, Dewey ME, Griffiths-Jones HM. A computerized psychiatric diagnostic system and case nomenclature for elderly subjects: GMS and AGECAT. *Psychol Med* 1986; 16(1):89-99.

(22) Lasserson DS. Initial management of suspected transient cerebral ischaemia and stroke in primary care: implications of recent research. *Postgrad Med J* 2009; 85(1006):422-427.

(23) Jones WJ, Williams LS, Meschia JF. Validating the Questionnaire for Verifying Stroke-Free Status (QVSFS) by neurological history and examination. *Stroke* 2001; 32(10):2232-2236.

(24) Wilson A, Potter J, Taub N, et al. The effectiveness of a modified version of the Wilkinson questionnaire in screening for TIA and minor stroke in the United Kingdom. *Age Ageing* 2005; 34(1):30-35.

(25) Murray S, Bashir K, Lees KR, et al. Epidemiological aspects of referral to TIA clinics in Glasgow. *Scott Med J* 2007; 52(1):4-8.

(26) Martin PJ, Young G, Enevoldson TP, et al. Overdiagnosis of TIA and minor stroke: experience at a regional neurovascular clinic. *QJM* 1997; 90(12):759-763.

For peer review only

(27) Boysen G, Jensen G, Schnohr P. Frequency of focal cerebral transient ischemic attacks during a 12 month period. The Copenhagen city heart study. *Stroke* 1979; 10(5):533-534.

(28) Johnson SE, Skre H. Transient cerebral ischemic attacks in the young and middle aged. A population study. *Stroke* 1986; 17(4):662-666.

(29) Amort M, Fluri F, Schafer J, et al. Transient ischemic attack versus transient ischemic attack mimics: frequency, clinical characteristics and outcome. *Cerebrovasc Dis* 2011; 32(1):57-64.

(30) Dawson J, Lamb KE, Quinn TJ, et al. A recognition tool for transient ischaemic attack. *QJM* 2009; 102(1):43-49.

(31) De Haan EH, Nys GM, van Zandvoort MJ. Cognitive function following stroke and vascular cognitive impairment. *Curr Opin Neurol* 2006; 19(6):559-564.

(32) Selnes OA, Vinters HV. Vascular cognitive impairment. *Nat Clin Pract Neurol* 2006; 2(10):538-547.

(33) Dennis MS, Bamford JM, Sandercock PA, et al. Incidence of transient ischemic attacks in Oxfordshire, England. *Stroke* 1989; 20(3):333-339.

(34) Rothwell PM, Coull AJ, Giles MF, et al. Change in stroke incidence, mortality, case-fatality, severity, and risk factors in Oxfordshire, UK from 1981 to 2004 (Oxford Vascular Study). *Lancet* 2004; 363(9425):1925-1933.

For peer review only

Table 1: Demographics of Participants in the Cognitive Function and Aging Study (CFAS) by sex, age, social class and comorbidity (Data are count (%))

	All (n=11903)		Males (n=4689)		Females (n=7214)	
	N	%*	N	%*	N	%*
**Age**						
65-74	5980	58.8	2635	64.5	3345	55.0
75-84	4624	32.1	1716	29.6	2908	33.8
85+	1299	9.1	338	5.9	961	11.2
**Social Class**						
I	558	4.8	225	4.8	333	4.8
II	2988	26.1	1213	26.3	1775	25.9
IIIa	1337	11.6	456	9.9	881	12.8
IIIb	4295	37.7	1865	40.8	2430	35.5
IV	1727	14.9	650	13.9	1077	15.7
V	561	4.9	196	4.3	365	5.3
**Comorbidity**						
Angina	1923	16.3	878	15.3	1045	11.1
Diabetes	672	5.5	310	6.4	362	4.9
Heart Attack	1139	9.5	632	13.6	507	6.8

*Weighted percentages

For peer review only

Table 2: Distribution and adjusted odds of transient neurological symptoms in those aged 65 or over in the Cognitive Function and Aging Study (CFAS) by sex, social class and comorbidity (Data are count (%))

	Loss of Speech			Weakness			Loss of Sight			At least one symptom		
	**N**	**%***	**OR † (95%CI)**	**N**	**%***	**OR† (95%CI)**	**N**	**%***	**OR† (95%CI)**	**N**	**%***	**OR† (95%CI)**
**Gender**												
Male	119	2.5	1.0	257	5.5	1.0	347	7.6	1.0	598	12.8	1.0
Female	152	2.2	0.9(0.7-1.2)	339	4.8	0.9(0.7-1.0)	525	7.6	1.0(0.9-1.2)	858	12.0	1.0 (0.9-1.1)
**Age**												
65-74	137	2.3	1.0	302	5.1	1.0	480	8.1	1.0	768	12.9	1.0
75-84	104	2.3	0.7(0.7-1.2)	229	5.0	0.9 (0.7-1.1)	327	7.2	0.9(0.7-1.1)	553	12.0	0.9 (0.8-1.0)
85+	30	2.5	0.8(0.5-1.3)	65	5.4	0.9(0.6-1.2)	65	5.4	0.6(0.5-0.8)	134	10.4	0.7 (0.6-0.9)
**Social Class**												
Non-manual	96	5.8	1.0	63	11.0	1.0	295	17.9	1.0	497	10.2	1.0
Manual	164	7.2	1.1 (0.9-1.5)	390	17.8	1.4 (1.2-1.7)	556	25.7	1.4(1.2-1.6)	920	14.1	1.4 (1.2-1.6)
**Comorbidity**												

For peer review only

Angina (n=1538)	64	4.3	1.5(1.0-2.2)	176	11.7	2.5(2.0-3.2)	160	10.6	1.3(1.0-1.6)	312	20.7	1.7(1.4-1.9)
Diabetes (n=672)	28	4.0	1.4(0.9-2.2)	40	6.1	1.0(0.7-1.4)	66	10.1	1.3(0.9-1.7)	102	15.2	1.1(0.9-1.4)
Heart Attack (n=1139)	52	4.7	1.8(1.2-2.7)	131	10.5	1.4(1.2-1.9)	119	11.5	1.4(1.1-1.8)	233	12.4	1.4(1.2-1.8)

* Weighted percentages

†Adjusted for age, sex, social class, cognition and cardiovascular comorbidities

‡ Reference non-manual (I,II, IIIa) compared to manual social class (IIIb,IV,V)

156 participants had missing data on the speech questionnaire, 155 on the weakness and 154 on the sight question

For peer review only

Table 3: Two year incidence of transient neurological symptoms in CFAS in those without stroke at baseline at two yr Screening Assessment, N at 2yrs= 6748 (Data are N (%))

	**Age**							
**Transient Neurological Symptom**	**All**		**65-74 N=3657**		**75-84 N=2618**		**85+ N=473**	
	**N**	**%***	**N**	**%***	**N**	**%***	**N**	**%***
Loss of Speech	149	2.1	55	1.5	84	3.3	10	2.0
Weakness	239	3.7	117	3.4	106	4.1	16	3.4
Loss of Sight	410	6.3	214	6.3	170	6.6	26	4.5
**At least one**	**675**	**9.8**	**327**	**9.3**	**304**	**11.6**	**44**	**7.8**

*Weighted percentage

98 participants had missing data on the speech questionnaire, 97 on the weakness and 105 on the sight question

For peer review only

Table 4: Epidemiological studies of self-reported prevalence of sudden neurological symptoms of weakness of limbs, loss of sight and loss of speech

Study	Age Range	Date	Population	N	Question	How	Frequencies

Table 4a: Frequencies of self-reported sudden weakness of limbs across population studies

For peer review only - http://bmjopen.bmj.com/site/about/guidelines.xhtml

Renfrew/ Paisley ***Hart et al. (2001)***[1]	45-64	1972-1976	General population study residents Renfrew&Paisley, Scotland Excluded those with stroke	1511 3	Have you ever without warning suddenly lost the power of an arm?	Questionnaire checked when attended a clinic	344 (2.3%)
Renfrew/ Paisley ***Hart et al. (2001)***[1]	45-64	1972-1976	General population study residents Renfrew&Paisley, Scotland Excluded those with stroke	1511 3	Have you ever without warning suddenly lost the power of a leg?	Questionnaire checked when attended a clinic	363 (2.4%)
Atherosclerosis Risk in Communities (ARIC) Cohort ***Toole et al (1996)*** [2]	45-64	1987-1989	Four centres in US adults residents	12,20 5	Have you ever had sudden painless weakness on one side of your body?	Administered by researcher – questions read aloud	283 (2.3%)
Baltimore population study ***Mules et al (1971)*** [3]	>40	1954-1967	Population samples – two in US	9937	Paralysis within 2 years prior to interview	Interview by trained interviewer	48(0.7%)
Reasons for geographic and racial differences in Stroke (REGARDS) ***Howard et al (2006)*** [4]	> 45 (mean 65)	2003	Community dwelling individuals in US. Those with stroke and TIA excluded	1846 2	Have you ever had sudden painless weakness on one side of the body?	QVSFS questionnaire – telephone and in person	1065 (5.8%)
Survey of Elderly Persons Living in Retirement Facilities ***Wilkinson et al (1979)*** [5]	>60	Published 1979	Elderly living in retirement facilities in 8 cities in the US	7281	During last **12 months** any sudden occurrence of transient episode of weakness or paralysis of limbs *which subsequently cleared up completely*	Self-administered questionnaire	197 (2.7%)

For peer review only - http://bmjopen.bmj.com/site/about/guidelines.xhtml

Table 4b: Frequencies of self-reported sudden speech impairment across population studies

Study	Age Range	Date	Population	N	Question	How	Frequencies
Renfrew/ Paisley ***Hart et al. (2001)***[1]	45-64	1972-1976	General population study residents Renfrew&Paisley, Scotland Excluded those with stroke	15113	Have you ever without warning suddenly been unable to speak properly?	Questionnaire checked when attended a clinic	270 (1.8%)
Atherosclerosis Risk in Communities (ARIC) Cohort ***Toole et al (1996)*** [2]	45-64	1987-1989	Four centres in US adults residents	12,205	Have you ever had sudden episode of "speech dysfunction"?	Administered by researcher – questions read aloud	313 (2.6%)
Reasons for geographic and racial differences in Stroke (REGARDS) ***Howard et al (2006)*** [4]	> 45 (mean 65)	2003	Community dwelling individuals in US. Those with stroke and TIA excluded	18462	Have you ever had sudden loss of ability to express self verbally (or in writing)?	QVSFS questionnaire – telephone and in person	698 (3.8%)
Survey of Elderly Persons Living in Retirement Facilities ***Wilkinson et al (1979)*** [5]	>60	Published 1979	Elderly living in retirement facilities in 8 cities in the US	7281	During last **12 months** experienced any sudden loss of speech or changes in speech *which subsequently cleared up completely*?	Self-administered questionnaire	149 (2.0%)

For peer review only - http://bmjopen.bmj.com/site/about/guidelines.xhtml

Table 4c: Frequencies of self-reported sudden visual impairment across population studies

Study	Age Range	Date	Population	N	Question	How	Frequencies
Atherosclerosis Risk in Communities (ARIC) Cohort ***Toole et al (1996)*** [2]	45-64	1987-1989	Four centres in US adults residents	12,205	Have you ever had sudden "visual impairment"?	Administered by researcher – questions read aloud	313 (2.6%)
Reasons for geographic and racial differences in Stroke (REGARDS) ***Howard et al (2006)*** [4]	> 45 (mean 65)	2003	Community dwelling individuals in US. Those with stroke and TIA excluded	18462	Have you ever had sudden painless loss of vision in one or both eyes?	QVSFS questionnaire – telephone and in person	698 (3.8%)
Baltimore population study ***Mules et al (1971)*** [3]	>40	1954-1967	Population samples – two in US	9937	Loss of vision within 2 years prior to interview	Interview by trained interviewer	129 (2.0%)
Survey of Elderly Persons Living in Retirement Facilities ***Wilkinson et al (1979)*** [5]	>60	Published 1979	Elderly living in retirement facilities in 8 cities in the US	7281	During last **12 months** experienced sudden loss of eyesight *which subsequently cleared up completely*?	Self-administered questionnaire	149 (2.0%)

For peer review only - http://bmjopen.bmj.com/site/about/guidelines.xhtml

(1) Hart CL, Hole DJ, Smith GD. The relation between questions indicating transient ischaemic attack and stroke in 20 years of follow up in men and women in the Renfrew/Paisley Study. J Epidemiol Community Health 2001; 55(9):653-656.

(2) Toole JF, Lefkowitz DS, Chambless LE, Wijnberg L, Paton CC, Heiss G. Self-reported transient ischemic attack and stroke symptoms: methods and baseline prevalence. The ARIC Study, 1987-1989. Am J Epidemiol 1996; 144(9):849-856.

(3) Mules J, Henderson M, Kuller L. A population survey of symptoms suggestive of transient ischemic attacks. Stroke 1971; 2(2):114-127.

(4) Howard VJ, McClure LA, Meschia JF, Pulley L, Orr SC, Friday GH. High prevalence of stroke symptoms among persons without a diagnosis of stroke or transient ischemic attack in a general population: the REasons for Geographic And Racial Differences in Stroke (REGARDS) study. Arch Intern Med 2006; 166(18):1952-1958.

(5) Wilkinson WE, Heyman A, Burch JG, Ostfeld A, Labarthe DR, Leviton A et al. Use of a self-administered questionnaire for detection of transient cerebral ischemic attack

For peer review only

Transient neurological symptoms in the older population: Report of a prospective cohort study - the Medical Research Council Cognitive Function and Ageing Study (CFAS)

Nahal Mavaddat, George M Savva, Daniel Lasserson, Matthew Giles, Carol Brayne, Jonathan Mant

Department of Public Health and Primary Care, University of Cambridge, Strangeways Laboratory, Worts Causeway, Cambridge, UK CB1 8RN Nahal Mavaddat clinical lecturer Jonathan Mant professor

~~The Irish Longitudinal Study on Ageing, Department of Gerontology, Trinity College Dublin, Lincoln Gate, Dublin 2, Republic of Ireland~~ School of Nursing Sciences, University of East Anglia, Norwich Research Park, Norwich, UK, NR4 7TJ. George Savva senior lecturer

Department of Primary Care Health Sciences, University of Oxford, Oxford, U.K. New Radcliffe House, 2nd floor, Walton Street, Jericho, UK OX2 6NW Daniel Lasserson clinical lecturer

Stroke Prevention Research Unit, NIHR Biomedical Research Centre, Oxford University, Department of Clinical Neurology, John Radcliffe Hospital, Oxford, UK OX3 9DU Matthew Giles consultant physician

Department of Public Health and Primary Care, University of Cambridge, Institute of Public Health, Forvie Site, Robinson Way Causeway, Cambridge, UK CB2 0SR Carol Brayne professor

Correspondence to: Nahal Mavaddat nm212@medschl.cam.ac.uk

The Corresponding Author has the right to grant on behalf of all authors and does grant on behalf of all authors, an exclusive licence (or non exclusive for government

employees) on a worldwide basis to the BMJ Publishing Group Ltd to permit this article (if accepted) to be published in BMJ editions and any other BMJPGL products and sublicences such use and exploit all subsidiary rights, as set out in our licence.

For peer review only

Article Summary

Article Focus:

- Prompt initiation of secondary prevention following TIA is associated with up to 80% reduction in risk of subsequent stroke.
- Many people presenting to specialist TIA clinics with transient neurological symptoms do not have TIA.
- The prevalence and incidence of transient neurological symptoms (as opposed to TIA) in older age groups is unknown.

Key Messages:

- In a large multi-centred community based study representative of the older population in the UK, we found a high prevalence and incidence of transient neurological symptoms, significantly greater than that of TIAs in hospital and population-based studies.
- The high rates of transient neurological symptoms in the community in the older population should be considered when planning TIA services. ~~highlights the need for adequate provision of TIA services and better diagnostic tools, as the public becomes more sensitised to possible cerebrovascular symptoms, and primary care practitioners more aware of the potential benefits of rapid response to suspected TIA.~~

Strengths and Limitations of this study:

- Strengths - Previous studies of incidence and prevalence of transient neurological symptoms have mostly been conducted in populations not

For peer review only - http://bmjopen.bmj.com/site/about/guidelines.xhtml

representative of older age groups where TIA is most common and few have been undertaken in UK populations.

- Limitations - The incidence of transient neurological symptoms in the study was determined in people without severe cognitive impairment, and may be an underestimate since cognitive impairment can be a manifestation of vascular disease. The wording of questions to identify transient neurological symptoms may have picked up some people who had symptoms lasting more than 24 hours. ~~Such patients are nevertheless likely to draw on TIA services for further assessment.~~

For peer review only

ABSTRACT

Objective: Transient ischaemic attack (TIA) is a recognised risk factor for stroke in the older population requiring timely assessment and treatment by a specialist. The need for such TIA services is driven by the epidemiology of transient neurological symptoms, which may not be caused by TIA. We report prevalence and incidence of transient neurological symptoms in a large UK cohort study of older people. **Design** Longitudinal cohort study **Setting and Participants:** The MRC Cognitive Function and Aging Study (CFAS) is a population representative study derived from random samples of people in their 65th year. Interview at baseline included questions about stroke and three transient neurological symptoms, repeated in a sub-sample after two years. Patients were flagged for mortality. **Main Outcome Measures** Prevalence and two year incidence of transient neurological symptoms. **Results** In 11,903 participants without a history of stroke, 271(2.3%) reported transient problems with speech, 872 (7.6%) with sight, and 596 (5.1%) weakness in a limb, with 1456 (12.7%) reporting at least one symptom. Of those re-interviewed (n=6748), 675 (9.8%) reported at least one symptom over two years. **Conclusions** Lifetime prevalence and incidence of transient neurological symptoms in people 65 and over is high and is substantially greater ~~higher~~ than the incidence of TIA in hospital and population-based studies. These high rates of transient neurological symptoms in the community in the older population should be considered when planning TIA services. ~~With increasing public education regarding stroke, TIA services may need to cope with an increase in demand as a greater proportion of people with transient neurological symptoms present for urgent assessment.~~

For peer review only

INTRODUCTION

Transient ischaemic attack (TIA) is an established and powerful risk factor for stroke. 8% of patients who have a TIA suffer a stroke within 7 days, many within 48 hours.[1;2] Immediate specialist assessment and treatment is associated with substantial reductions in this early risk of stroke, [3] and is recommended for people with suspected TIA. [4]

As awareness grows of the urgency of early management of TIA amongst both primary care practitioners and the general public, [5] more patients with symptoms suggestive of a TIA are likely to present to primary and secondary care services. Need for specialist services will be driven by incidence of symptoms that might represent TIA (i.e. transient neurological symptoms) rather than the epidemiology of TIA *per se*. Currently about 48,000 probable or definite TIAs (transient neurological symptoms lasting less than 24 hours of likely vascular aetiology), and 43,000 minor strokes (stroke events causing minimal or no neurological deficits) are managed as outpatients every year in England, but it is not clear to what extent this might be the tip of the iceberg. [6] Data are available worldwide on the prevalence and incidence of TIA. [7-11] The epidemiology in the community of transient neurological symptoms (i.e. neurological symptoms of sudden onset of which TIA is a subset), is however less well defined. Previous studies of incidence and prevalence of transient neurological symptoms, have mostly been conducted in populations not representative of older age groups where TIA is most common. [7;11-16] Very few have been undertaken in UK populations. While incidence of transient neurological symptoms will drive need for specialist TIA services, prevalence of transient neurological symptoms in the

For peer review only - http://bmjopen.bmj.com/site/about/guidelines.xhtml

community is also important. TIA is associated with a long term increase in risk of stroke, [17;18] so there may be value in diagnosing 'old' events that have not presented to medical services in order to target secondary prevention. Hence the need to know both the incidence and prevalence of transient neurological symptoms in the community.

This study reports prevalence and incidence of three common transient neurological symptoms (limb weakness, loss of speech and disturbance of vision) in a population based multi-centred cohort study in the UK (England and Wales) in those aged 65 and over, the Medical Research Council Cognitive Function and Ageing Study (CFAS). [19]

METHODS

The Medical Research Council CFAS is a population based representative study of individuals aged 65 years and over living in the community. The study began in 1991 and was designed to determine the incidence of dementia in the older population. It has six centres across England and Wales chosen to represent the national variation of urban-rural mix, socio-economic deprivation and rates of chronic disease.[19] Five of these with identical study designs (Oxford, Nottingham, Cambridgeshire Gwynedd and Newcastle) are used in the present investigation. The sixth centre (Liverpool) used a different design and is therefore excluded. Random samples of people in their 65th year and above were obtained from Family Health Service Authority lists (agency responsible for maintaining registers of general practice populations at that time). The sample was stratified by age (65-74, and 75 years and over) and equal numbers were randomly selected from these groups with the aim of recruiting 2500 to each centre. All study centres obtained ethical approval from local research

For peer review only - http://bmjopen.bmj.com/site/about/guidelines.xhtml

committees. (REC Ref: 05/MRE05/37). Full details of methodology are available elsewhere. [19]

Eligible participants (or their proxies where appropriate) provided informed consent ~~(or their proxies where appropriate)~~. Trained interviewers undertook baseline interviews in the participants' homes including socio-demographic characteristics, cognitive function and disease history including previous stroke, coronary heart disease and diabetes (full details at www.cfas.ac.uk). Based on baseline screening, the study sample was divided into two groups at baseline: people without cognitive impairment and a group consisting of those with cognitive impairment plus a stratified subsample of those without cognitive impairment. The first group underwent no further assessment at baseline and were 'rescreened' after two years. The second group underwent a further detailed cognitive assessment at baseline, but were not followed up for transient neurological symptoms. The estimates of prevalence of transient neurological symptoms are calculated for all people at baseline, while the incidence estimates use only the first group. All participants were flagged with the NHS Central Register. Deaths and underlying causes of death attributed to stroke (ICD codes 430-438) were notified to the study.

Prevalence of transient neurological symptoms

At baseline all participants were asked "Have you ever experienced sudden problems with:

(1) speech, which got better after a day?

(2) weakness in the arms or legs, which got better after a day?

(3) sight, which got better after a day?"

For peer review only - http://bmjopen.bmj.com/site/about/guidelines.xhtml

Social class was determined using the Registrar General's Occupational Classification. Cognitive status was determined using the Mini Mental State Examination (MMSE), [20] and the Automated Geriatric Examination for Computer Assisted Taxonomy (AGECAT). [21]

Incidence of transient neurological symptoms

All those in the 'rescreen' arm were asked at two year follow-up if they had experienced each transient neurological symptom in the past two years.

Analysis

All analyses were carried out using STATA version 11. Inverse probability weights were used throughout to ensure the sample was representative of the target population. Weights were estimated with logistic regression using presence in each phase of the study as an outcome and taking into account over-sampling of over-75s at baseline. Weights for incidence calculations adjust for attrition based on baseline characteristics and stratified selection into the assessment arm as appropriate. Baseline prevalence of each transient neurological symptom was calculated for age specific strata and as a weighted percentage to provide a population estimate of prevalence for people aged 65 and over. Associations with gender, age, social class (manual (IIIb, IV, V) and non-manual (I, II, IIIa)), and cardiovascular comorbidity were explored using logistic regression models adjusting for all other factors and cognitive function based on MMSE by score (less than 18, 18-21, 22-25 and 26-30).

Two year incidence of transient neurological symptoms was estimated using weighted percentages of those reporting any of the symptoms during follow-up. Weights were adjusted for refusals, drop-outs and for non-reassessment of people with cognitive

For peer review only - http://bmjopen.bmj.com/site/about/guidelines.xhtml

impairment. Calculation of attrition weights for incident transient neurological symptoms excluded those who died between baseline and follow up. These estimates are therefore applicable to the population over 65 without severe cognitive impairment or stroke at baseline surviving at least two years.

RESULTS

The participation rate in the CFAS study was 80% (13,004/16,258). For the prevalence analysis, participants who had a stroke at baseline (963, 7.4%) or for whom baseline information about stroke was missing (138, 1.1%) were excluded, leaving 11903 participants. 2,283 of the original participants were not allocated to the 'rescreen' arm at two years; 754 died, 1973 declined to participate and 145 were lost to follow-up, leaving 6,748 (76% response rate of potential participants) for the incidence analysis.

Table 1 shows the demographic features of participants in the CFAS studied in this analysis at baseline. 40% of participants were male and 60% of participants were female. 12.3% of all participants reported at least one transient neurological symptom. 2.3% reported transient loss of speech, 5.1% transient weakness and 7.6% transient loss of sight.

Table 2 shows prevalence data with weighted percentages and odds ratios (adjusted for age, sex, social class, cognition and cardiovascular morbidities) of reported transient neurological symptoms at baseline by demographic factors and comorbidities. 12.8% of males and 12.0% of females reported at least one of the three transient neurological symptoms of loss of speech, loss of sight, or weakness. There was no significant association of gender with reporting at least one transient

For peer review only

neurological symptom, but significantly lower odds of reporting a transient neurological symptom in the over 85 age group. Problems of memory and recall may have contributed to under-reporting in this group. Greater rates of mortality associated with neurological incidents in the oldest old population may also lead to under estimation of rates of transient neurological symptoms. The odds of reporting any transient neurological symptom were higher in those in manual compared to non-manual social classes. The presence of cardiovascular morbidities of angina and heart attack were also significantly associated with the odds of having at least one of the transient neurological symptoms.

There was no difference between genders with regards to reporting individual transient neurological symptoms of loss of speech, loss of sight or weakness. However, there was a significantly lower odds of reporting transient visual loss in those over 85, and. This may partly explain the overall lower prevalence of reporting at least one transient neurological symptom in this group. The cause for this is unclear, however in addition to problems of recall, may in part relate to the greater likelihood of chronic visual problems potentially masking transient visual losses in the oldest old population. an increased odds of reporting transient symptoms of weakness and loss of sight in manual compared to non-manual social classes. The presence of cardiovascular co-morbidities of angina and heart attack were associated with higher odds of all three symptoms.

Table 3 shows two year incidence of transient neurological symptoms in respondents attending the “rescreen”. 9.8% of all participants reported at least one transient neurological symptom over the 2 years, with the highest incidence of reported

For peer review only - http://bmjopen.bmj.com/site/about/guidelines.xhtml

transient neurological symptom being due to loss of sight followed by weakness, with loss of speech being the least frequently reported transient neurological symptom. The incidence of each of the transient neurological symptoms was highest in the 75 to 84 age group and lowest in the over 85 age group.

DISCUSSION

Our findings suggest that transient neurological symptoms are common in the older population in England and Wales, with at least 12% of people aged 65 and over having experienced a transient neurological symptom of the arm or leg, speech or vision better after a day, and approximately 5% experiencing at least one such symptom over the course of a year. The commonest of these was transient symptoms of vision, followed by limb weakness. Those over 85 reported lower rates of transient neurological symptoms, predominantly due to less frequent reporting of transient visual loss. Problems of memory and recall may have contributed to under-reporting, and higher mortality rates associated with neurological incidents to underestimation of rates of transient neurological symptoms in this age group. Chronic visual problems may have also potentially masked transient visual losses in the oldest-old population. Incidence of at least one transient neurological symptom in the CFAS population was approximately 2.6 times greater than that of confirmed TIA presenting to medical services in the Oxford Vascular Study (OxVASC) in people aged over 85 (approximately 15 per 1000 per year), 8 times greater in those aged 75 to 84 (7 per 1000 per year), and approximately 15 times greater in those aged 65 to 74 (3 per 1000 population per year). [10]

For peer review only - http://bmjopen.bmj.com/site/about/guidelines.xhtml

The prevalence and incidence of transient neurological symptoms in the CFAS was somewhat higher than those found by questionnaires used in other studies internationally (mostly conducted in younger age groups), but comparable to those of Wilkinson et al. in an over 60 age group in a US based study. [7;12-14;16] Table 4 (online) shows the prevalence of sudden onset of neurological symptoms of weakness in a limb or of loss of speech or sight in previous population and community studies. [7;12-14;16] Questions used to elicit transient neurological symptoms in the CFAS are likely to have captured greater numbers of transient neurological events in the population compared to those seeking more specific vascular symptoms, or to the few studies defining more precisely the onset, offset and timing of the event. [7;12-14] While some respondents will have been describing true transient ischaemic events, it is likely that many did not have a true TIA. Validated measures to determine the presence of previous transient ischaemic attack are limited, and most studies of transient neurological symptoms in questionnaires overestimate true transient ischaemic attacks. [13;14;22] Wilkinson et al. (1979) suggest that around 10% of transient symptoms reported in a questionnaire are subsequently diagnosed as TIA after neurologist assessment.[14] Self-reported transient neurological symptoms in other community based studies have low positive predictive value, with higher values only in studies in outpatient populations (over 70%). [13;14;22-24] This suggests that the use of screening to identify possible past transient ischaemic attacks may generate unnecessary extra strain on primary care and secondary TIA services with limited benefit.

Patients presenting with transient neurological symptoms present considerable diagnostic dilemmas for primary care practitioners reflected in low rates of TIA diagnosis confirmation in patients referred to UK TIA clinics (over 50% being for

For peer review only

non-TIA causes).[6;25;26] Many symptoms of TIA are non-specific and occur in non-vascular syndromes. Dizziness, ophthalmological problems, migraine, epilepsy, nerve entrapment or psychological states are the commonest non-TIA diagnoses.[25-29] Often, however, no diagnosis can be determined.[25;26] Scales such as the ABCD2[2] help determine the urgency of TIA referral, but do not distinguish TIA from non-TIA symptoms. Wilkinson et al. reported transient loss of speech and loss of sight to be more reliable than limb weakness for neurologist diagnosis of TIA,[14] while Hart et al. found the strongest and most consistent relationship with subsequent stroke to be loss of power in an arm.[7] Other studies suggest that the presence of certain symptoms such as headache, dizziness, loss of consciousness, memory loss, blurred vision, generalised weakness, pain in limbs and seizures make the diagnosis of TIA less likely.[14;29;30]

Study limitations

The incidence of transient neurological symptoms ~~in the CFAS~~ was determined in people without severe cognitive impairment, and may be an underestimate since cognitive impairment can be a manifestation of vascular disease.[31;32] Only three transient neurological symptoms were reported in this study. Some symptoms of TIA (e.g. posterior circulation or pure sensory symptoms) were not sought ~~in the CFAS~~. The wording of questions to identify transient neurological symptoms may have picked up some people who had symptoms lasting more than 24 hours. Such patients are nevertheless likely to draw on TIA services for further assessment. In addition, it was not possible to determine whether different transient neurological symptoms in a participant occurred simultaneously or separately in time. The problem of recall bias in these self-reports of transient symptoms needs to be considered. Questions were

For peer review only - http://bmjopen.bmj.com/site/about/guidelines.xhtml

designed to be easily comprehensible to the older population. However, the need to consider multiple criteria when responding to a question e.g. "sudden onset" and "less than a day", may have led to difficulties in interpretation of the transient neurological symptoms questions for some participants. Finally, baseline and two year follow-up were carried out in the 1990s and changes in rates of vascular events may have occurred since then. While the age-specific incidence of stroke appears to be declining, it is not clear that the same is true of TIA. [10;33;34] As well, this study is more recent than many studies estimating rates of transient neurological symptoms in the population, many of which have been carried out during the 1970's or '80's. [12-14]

Conclusion

In a large multi-centred community based study highly representative of the older population in the UK and conducted in the age group where TIAs are most common, we found a high prevalence and incidence of transient neurological symptoms. The incidence of such symptoms in the community is significantly greater than that of TIAs. ~~, suggesting that potential demand for TIA clinics where a confirmation of TIA diagnosis is made by a specialist, might rise significantly in the future as the public becomes more sensitised to possible cerebrovascular symptoms, and primary care practitioners more aware of the potential benefits of rapid response to suspected TIA.~~ This highlights the need for adequate provision of TIA services and the potential importance of the development of valid diagnostic tools to assist the general practitioner and hospital doctor in better triage of people presenting with transient neurological symptoms.

ACKNOWLEDGEMENTS

We thank the MRC CFAS study group for data collection and management. We are also grateful to all respondents, their families and their primary care teams from across the country for their participation in ~~the~~ MRC CFAS.

COMPETING INTERESTS

"All authors have completed the ICMJE uniform disclosure form at www.icmje.org/coi_disclosure.pdf and declare: no support from any organisation for the submitted work; no financial relationships with any organisations that might have an interest in the submitted work in the previous three years; no other relationships or activities that could appear to have influenced the submitted work."

FUNDING

The MRC CFAS Study has had funding by major awards from the Medical Research Council (MRC/G9901400) and Department of Health. NM was funded by a National Institute for Health Research (NIHR) Walport Clinical Lectureship in Primary Care Research. None of the sponsors influenced the design or conduct of the study or the analysis or interpretation of the findings. All researchers acted independently from funders in the design and carrying out of this study.

AUTHORS CONTRIBUTIONS

CB is Principal Investigator of the MRC CFAS Study. NM and GS contributed to analysis of data. All authors contributed to study design and intellectual input. All authors, external and internal, had full access to all of the data (including statistical

For peer review only

reports and tables) and can take responsibility for the integrity of the data and accuracy of the data analysis.

DATA SHARING

Data sharing through application.

For peer review only

Reference List

(1) Coull AJ, Lovett JK, Rothwell PM. Population based study of early risk of stroke after transient ischaemic attack or minor stroke: implications for public education and organisation of services. *BMJ* 2004; 328(7435):326.

(2) Rothwell PM, Giles MF, Flossmann E, Lovelock CE, Redgrave JN, Warlow CP et al. A simple score (ABCD) to identify individuals at high early risk of stroke after transient ischaemic attack. *Lancet* 2005; 366(9479):29-36.

(3) Rothwell PM, Giles MF, Chandratheva A, Marquardt L, Geraghty O, Redgrave JN et al. Effect of urgent treatment of transient ischaemic attack and minor stroke on early recurrent stroke (EXPRESS study): a prospective population-based sequential comparison. *Lancet* 2007; 370(9596):1432-1442.

(4) Department of Health. Department of Health. National Stroke Strategy. 2007.

(5) Department of Health. Stroke: Act F.A.S.T. awareness campaign. 2009. Department of Health.

(6) Giles MF, Rothwell PM. Substantial underestimation of the need for outpatient services for TIA and minor stroke. *Age Ageing* 2007; 36(6):676-680.

(7) Hart CL, Hole DJ, Smith GD. The relation between questions indicating transient ischaemic attack and stroke in 20 years of follow up in men and women in the Renfrew/Paisley Study. *J Epidemiol Community Health* 2001; 55(9):653-656.

(8) Mant J, Wade DT, Winner S. Health care needs assessment: stroke. In: Stevens A, Raftery J, Mant J, Simpson S, editors. Health care needs assessment: the epidemiologically based needs assessment reviews. 2nd ed. Oxford: Radcliffe Medical Press; 2004. 141-244.

(9) Orlandi G, Gelli A, Fanucchi S, Tognoni G, Acerbi G, Murri L. Prevalence of stroke and transient ischaemic attack in the elderly population of an Italian rural community. *Eur J Epidemiol* 2003; 18(9):879-882.

(10) Rothwell PM, Coull AJ, Silver LE, Fairhead JF, Giles MF, Lovelock CE et al. Population-based study of event-rate, incidence, case fatality, and mortality for all acute vascular events in all arterial territories (Oxford Vascular Study). *Lancet* 2005; 366(9499):1773-1783.

(11) Bos MJ, van Rijn MJ, Witteman JC, Hofman A, Koudstaal PJ, Breteler MM. Incidence and prognosis of transient neurological attacks. *JAMA* 2007; 298(24):2877-2885.

(12) Mules J, Henderson M, Kuller L. A population survey of symptoms suggestive of transient ischemic attacks. *Stroke* 1971; 2(2):114-127.

For peer review only

(13) Toole JF, Lefkowitz DS, Chambless LE, Wijnberg L, Paton CC, Heiss G. Self-reported transient ischemic attack and stroke symptoms: methods and baseline prevalence. The ARIC Study, 1987-1989. *Am J Epidemiol* 1996; 144(9):849-856.

(14) Wilkinson WE, Heyman A, Burch JG, Ostfeld A, Labarthe DR, Leviton A et al. Use of a self-administered questionnaire for detection of transient cerebral ischemic attacks: I. Survey of elderly persons living in retirement facilities. *Ann Neurol* 1979; 6(1):40-46.

(15) Bots ML, van der Wilk EC, Koudstaal PJ, Hofman A, Grobbee DE. Transient neurological attacks in the general population. Prevalence, risk factors, and clinical relevance. *Stroke* 1997; 28(4):768-773.

(16) Howard VJ, McClure LA, Meschia JF, Pulley L, Orr SC, Friday GH. High prevalence of stroke symptoms among persons without a diagnosis of stroke or transient ischemic attack in a general population: the REasons for Geographic And Racial Differences in Stroke (REGARDS) study. *Arch Intern Med* 2006; 166(18):1952-1958.

(17) van Wijk I, Kappelle LJ, van GJ, Koudstaal PJ, Franke CL, Vermeulen M et al. Long-term survival and vascular event risk after transient ischaemic attack or minor ischaemic stroke: a cohort study. *Lancet* 2005; 365(9477):2098-2104.

(18) Clark TG, Murphy MF, Rothwell PM. Long term risks of stroke, myocardial infarction, and vascular death in "low risk" patients with a non-recent transient ischaemic attack. *J Neurol Neurosurg Psychiatry* 2003; 74(5):577-580.

(19) Brayne C, McCracken C, Matthews FE. Cohort profile: the Medical Research Council Cognitive Function and Ageing Study (CFAS). *Int J Epidemiol* 2006; 35(5):1140-1145.

(20) Folstein MF, Folstein SE, McHugh PR. "Mini-mental state". A practical method for grading the cognitive state of patients for the clinician. *J Psychiatr Res* 1975; 12(3):189-198.

(21) Copeland JR, Dewey ME, Griffiths-Jones HM. A computerized psychiatric diagnostic system and case nomenclature for elderly subjects: GMS and AGECAT. *Psychol Med* 1986; 16(1):89-99.

(22) Lasserson DS. Initial management of suspected transient cerebral ischaemia and stroke in primary care: implications of recent research. *Postgrad Med J* 2009; 85(1006):422-427.

(23) Jones WJ, Williams LS, Meschia JF. Validating the Questionnaire for Verifying Stroke-Free Status (QVSFS) by neurological history and examination. *Stroke* 2001; 32(10):2232-2236.

(24) Wilson A, Potter J, Taub N, Moore A, Robinson T. The effectiveness of a modified version of the Wilkinson questionnaire in screening for TIA and minor stroke in the United Kingdom. *Age Ageing* 2005; 34(1):30-35.

For peer review only

For peer review only - http://bmjopen.bmj.com/site/about/guidelines.xhtml

(25) Murray S, Bashir K, Lees KR, Muir K, MacAlpine C, Roberts M et al. Epidemiological aspects of referral to TIA clinics in Glasgow. *Scott Med J* 2007; 52(1):4-8.

(26) Martin PJ, Young G, Enevoldson TP, Humphrey PR. Overdiagnosis of TIA and minor stroke: experience at a regional neurovascular clinic. *QJM* 1997; 90(12):759-763.

(27) Boysen G, Jensen G, Schnohr P. Frequency of focal cerebral transient ischemic attacks during a 12 month period. The Copenhagen city heart study. *Stroke* 1979; 10(5):533-534.

(28) Johnson SE, Skre H. Transient cerebral ischemic attacks in the young and middle aged. A population study. *Stroke* 1986; 17(4):662-666.

(29) Amort M, Fluri F, Schafer J, Weisskopf F, Katan M, Burow A et al. Transient ischemic attack versus transient ischemic attack mimics: frequency, clinical characteristics and outcome. *Cerebrovasc Dis* 2011; 32(1):57-64.

(30) Dawson J, Lamb KE, Quinn TJ, Lees KR, Horvers M, Verrijth MJ et al. A recognition tool for transient ischaemic attack. *QJM* 2009; 102(1):43-49.

(31) De Haan EH, Nys GM, van Zandvoort MJ. Cognitive function following stroke and vascular cognitive impairment. *Curr Opin Neurol* 2006; 19(6):559-564.

(32) Selnes OA, Vinters HV. Vascular cognitive impairment. *Nat Clin Pract Neurol* 2006; 2(10):538-547.

(33) Dennis MS, Bamford JM, Sandercock PA, Warlow CP. Incidence of transient ischemic attacks in Oxfordshire, England. *Stroke* 1989; 20(3):333-339.

(34) Rothwell PM, Coull AJ, Giles MF, Howard SC, Silver LE, Bull LM et al. Change in stroke incidence, mortality, case-fatality, severity, and risk factors in Oxfordshire, UK from 1981 to 2004 (Oxford Vascular Study). *Lancet* 2004; 363(9425):1925-1933.

For peer review only

Published by Oxford University Press on behalf of the International Epidemiological Association
International Journal of Epidemiology 2006;35:1140–1145
doi:10.1093/ije/dyl199
© The Author 2006; all rights reserved. Advance Access publication 15 September 2006

COHORT PROFILE

Cohort Profile: The Medical Research Council Cognitive Function and Ageing Study (CFAS)†

Carol Brayne,[1]* Cherie McCracken[2] and Fiona E Matthews[3]

Origin of the study

Global ageing is a recent phenomenon. Its potential impact on social and economic aspects of more affluent countries highlighted ageing as a sufficiently important issue towards which to direct resources. Discussion between the Department of Health, Medical Research Council, and experts from the scientific and medical communities resulted in the decision that brain changes, most particularly cognitive decline, dementia, and their relation to disability were key topics requiring investigation at the population level. This prompted a decision to invest in research into this area and a working group was convened, which included those with epidemiological and biostatistical expertise relevant to such investigation. Out of this working group a successful bid for the study now known as the MRC Cognitive Function and Ageing Study emerged.

Study design

The study is a six-centre multidisciplinary multiphased longitudinal design (see map, Figure 1). There are five identical sites and one with a different sampling and interview structure. This centre (Liverpool) was already funded at the time of the discussions noted above and thus started earlier than the other five centres.[1] The other five centres (Cambridgeshire, Gwynedd, Newcastle, Nottingham, and Oxford) were able to follow a standardized design and are referred to as the five identical sites. Their basic structure was a two-phase design with a screening interview followed by an assessment interview shortly afterwards, with a repeat at 2 years. The fieldwork began in 1991.[2] There are many additional features, which are more fully described on the website (see below).

The aims of the study

The aims of the study have evolved over its existence and cover a wide range including descriptive epidemiology, neuropathology, policy, molecular epidemiology, and ethics.

The main descriptive epidemiological aims include (i) the estimation of the prevalence and incidence of cognitive decline and dementia, and geographical variation in those rates; (ii) the determination of the natural history of dementia, in particular the rate of progression of cognitive decline including the distribution of the interval between the identification of cognitive impairment and death, and (iii) the identification of factors associated with differing rates of cognitive decline and with the risk of dementia.

The principal neuropathological aim was to determine the contribution of different underlying pathologies to the rates of dementia and the geographic variation in these rates and to the burden of disability. Additional aims included to: (i) determine the prevalence and severity of pathological lesions in the brain of an unselected cohort of older people with and without cognitive impairment; (ii) determine the frequency of specific pathological diagnoses in people with cognitive impairment, and (iii) correlate severity of specific pathologies with patterns of cognition, function, and behaviour in life independently of clinical and pathological diagnostic categories.

The core aim related to policy was the evaluation of the degree of disability associated with cognitive decline and impairment, and the service needs this disability generates. These needs were to be compared with the needs generated by physical impairment. The study also sought to form the basis for longer-term studies of trends over time and by birth cohort of the prevalence and incidence of cognitive decline. In addition to these aims the breadth of the data collected has allowed the study to incorporate the investigation of expectation of life in various states of health, depression, and depressive symptomatology in the older population.

The DNA resource has been incorporated in a later phase of the study. The main molecular epidemiological aim has been to support genetics studies that have sought genes associated with all dementia, Alzheimer's disease, mixed and vascular dementia, cognitive impairment and decline.

A later aim of the study was to explore the ethical and legal aspects of brain donation within a population-based sample given changing perception surrounding organ donation.

The study also aimed to act as a core resource and provide a framework to support specific sub-studies in lone or joint centres. The Resource Implication Study[3–9] utilized this framework to achieve the core policy aim (see above). Other

[1] Department of Public Health and Primary Care, Institute of Public Health, Cambridge University, Forvie Site, Robinson Way, Cambridge CB2 2SR, UK.

[2] Department of Psychiatry, University of Liverpool University, Clinical Dept Duncan Building Liverpool L69 3BX, UK.

[3] MRC Biostatistics Unit, Institute of Public Health, Forvie Site, Robinson Way, Cambridge CB2 2SR, UK.

* Corresponding author. E-mail: Carol.Brayne@medschl.cam.ac.uk

† A full list of the study investigators and contributors is available on the website.

Downloaded from http://ije.oxfordjournals.org/ at Cambridge University Library on March 12, 2013

For peer review only - http://bmjopen.bmj.com/site/about/guidelines.xhtml

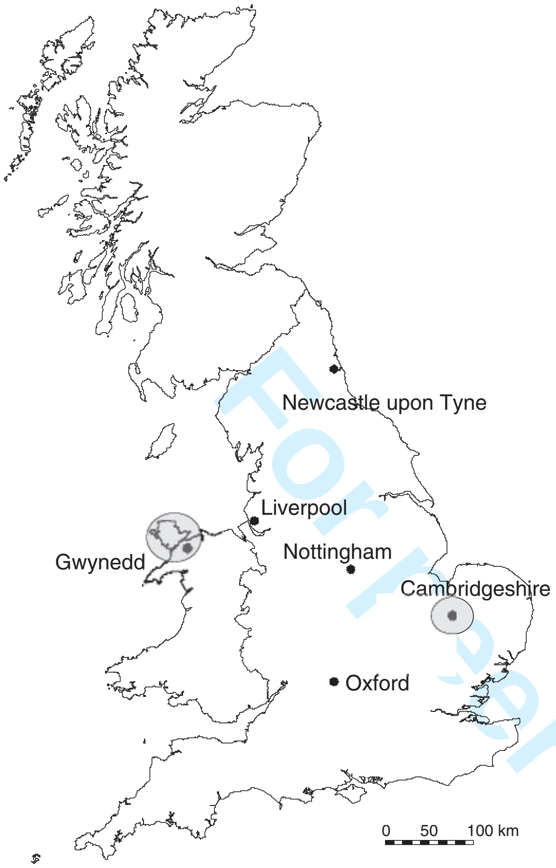

Figure 1 Map of Great Britain showing CFAS centres

sub-studies include the study of Healthy Ageing[10–15] and the Network Study. This framework also extends to wider collaborations with CFAS centres contributing to European wide initiatives such as EURODEM[16] and EURODEP.[17]

What does it cover?

Because the main study is focused on cognition and dementia it has collected the necessary dimensions of physical and mental health to arrive at a study diagnosis of dementia. It now has four major themes: (i) dementia (covering all aspects including cognition), (ii) depression, (iii) disability and healthy life expectancy, and (iv) health policy and health. In addition it has particular strengths in that it is one of the very few truly population based programmes with a donation programme—individuals in the study have indicated whether they wish to contribute to brain research through the donation of their brain after death (declaration of intention to donate).

Who is in the sample?

The first aim of the study was to estimate age-specific rates of prevalence of cognitive impairment and dementia among those aged 65 and over. The population is thus all those aged 65 years and over on the index date for centre (1990, 1991), living within a specified geographical location. Background information on the demographics of the populations sampled was collected from the Office of Population Census and Surveys (OPCS), 1990–91 census now Office of National Statistics (ONS), to relate to regional and national data.

Family Health Service Authority (FHSA) lists were used as the sampling frame. The frame would be incomplete if eligible members of the population were not registered with a GP. However individuals in long-stay hospitals remain registered with their GP 2 years after institutionalization so sampling from FHSA lists ensured their inclusion. Each centre looked into the practices of long-stay hospitals in their area to confirm this. The FHSA list of individuals was used for sampling on a geographical basis. Each centre defined this area, and the study population was drawn from all those who were resident within it. Problems of inaccuracy, patients who died or moved away but were still on the FHSA list, were resolved by asking GP surgeries to check the lists. On this basis, a sample of sufficient size to yield 2500 interviews of individuals aged 65 years and over, stratified by age (equal numbers aged 65–74 and 75 plus) was chosen from the FHSA lists for each selected area (in Liverpool this was 5000 interviews stratified by sex and 5 year age band). The population is flagged at ONS for mortality and the database is updated continuously.

The follow-up has been determined by funding and the design of associated bolt-on studies. The main follow-up waves for the identical sites are captured in the audit trail shown in Figure 2, which shows the numbers for the main screen, assessment, 1 year follow-up and 2 year rescreen, new selection for assessment and further 1 year follow-up, 6 year follow-up of the assessed (with venepuncture), 8 year follow-up of those with intentions to donate, and 10 year follow-up of the total sample. In addition to this the main associated studies are the Resource Implication Study (4 centres—Cambridgeshire, Newcastle, Nottingham, Oxford), which followed those who provided care to the physically and cognitively frail at baseline, the ESRC funded Healthy Ageing Project, which interviewed in detail those who were not selected into the Resource Implication Study in Nottingham and Cambridgeshire, the Network Study conducted in Gwynedd and Liverpool to examine individuals' social networks, an embedded case–control study at 2 year incidence stage (Cambridgeshire), and the ongoing brain donation programme in all centres. This programme, in combination with the bloods taken at year 6 form the major components of the Biological Resource of the study.

Who is not in the sample?

Comprehensive analyses of those who were lost to follow-up have been conducted for all stages of the study. At baseline 19% of potential respondents refused, 6% had died, and 1% had moved out of the area. Similar percentages were found for all waves of the study. Individuals who had moved or refused had higher mortality than responders.[18] CFAS has used this attrition for a detailed investigation of attrition effects in both short and longer time intervals.[18,19]

For peer review only

Downloaded from http://ije.oxfordjournals.org/ at Cambridge University Library on March 12, 2013

For peer review only - http://bmjopen.bmj.com/site/about/guidelines.xhtml

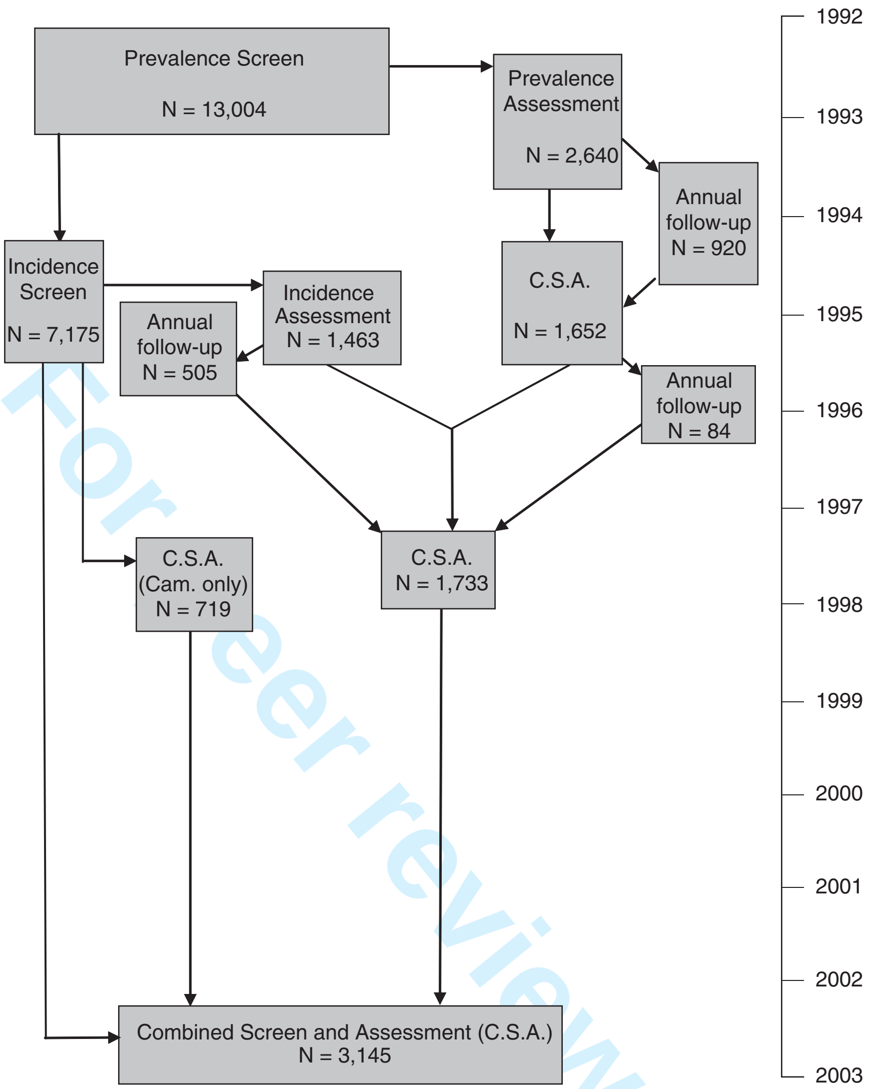

Figure 2 Time frame of CFAS interviews (identical centres)

For peer review only

How, when, and which interviews were conducted?

All interviews were conducted in the respondent's place of residence, using portable computers with customized software. If the interviewer felt that the respondent was frail and tiring, or becoming agitated, the short 'priority mode' set of questions could be invoked manually. Screening interviews were undertaken by lay interviewers, recruited for the purpose and trained by both the local and national coordinator. Reliability checks were made by both the local and the national coordinator. Proxy screening interviews were conducted where an interview was not possible with the named participant, owing to, for example, extreme confusion or frailty. If after four attempts to contact, an interview was not arranged, the approach was abandoned. The screening of the entire sample took 2 years to complete.

The assessment (designed to be conducted 1 month after the screening interview) and annual (designed to be conducted 1 year after the screening interview) interviews were also undertaken using customized portable computers. These interviews were carried out by interviewers from professions allied to medicine, who had not undertaken the screening interview, also recruited and trained for the purpose. Interviewers did not know the outcome of the first interview. The interviews lasted from 45 to 90 min, again with a 'priority mode' route. The annual interview consisted of a combined screen and assessment, where information on changes since last interview was recorded. At the assessment and biannual follow-up interview permission was sought to approach and interview a relative or carer to ask for an objective account of the respondent's health and abilities.

Six years after the initial screening interview, all respondents in the assessed groups were interviewed using the combined screen and assessment interview and at the end of that interview signed permission was requested to take a sample of blood or saliva. Permission was also sought to access GP and hospital notes. At 8 years only those who had indicated an

Downloaded from http://ije.oxfordjournals.org/ at Cambridge University Library on March 12, 2013

For peer review only - http://bmjopen.bmj.com/site/about/guidelines.xhtml

intention to donate brain tissue were re-interviewed with the combined screen and assessment interview.

At 10 years all survivors from the responding group of the complete study were recontacted for interview and if they agreed were interviewed using the combined screen and assessment interview.

What is collected at different interviews?

The screening interview contains questions on residence, marital status, education and occupation, living circumstances, contact with friends and family, health and social care contact, self-reported physical health, instrumental activities of daily living and activities of daily living, cognitive measures (Mini Mental State Examination with augmentation), and medication.

The assessment interview is mainly the Geriatric Mental State Examination (GMS) adapted for CFAS.[20] This is a structured psychiatric interview, which collects sufficient information for algorithmic 'diagnosis' in the major psychiatric disorders of old age (dementia, depression, anxiety, and psychosis). This has been validated against clinical diagnosis and the instrument has been widely used in Europe and now forms part of the 10/66 international instrument. This interview has been augmented with questions from the CAMDEX (Cambridge Examination for Mental Disorders in the Elderly) including CAMCOG,[21] the longer neuropsychological assessment. The relative or carer interview is mainly the History and Aetiological Schedule, the informant interview that accompanies the GMS.

The combined screen and assessment interview merges the two interviews but compresses some aspects of data collection. Complete versions of all the interviews including the interview questions and responses are available on the website.

The neuropathological assessment follows the standardized protocol of the Consortium to Establish a Registry for Alzheimer's Disease (CERAD)[22], with the exception that the neuropathologist is blind to the interview data. This covers in a semiquantitative form the main areas required for the assessment of neurodegenerative and cerebrovascular disorders. The forms are available on the website. The main genetic analyses have been on apoE and ACE.

Data collection in Liverpool was broadly similar to the other five sites except that the screening interview consisted of the GMS plus some of the screening interview questions listed above.

What has the study found?

The study has reported on prevalence and incidence of dementia, and lack of variation in these across the five identical sites.[23–25] It has provided profiles of cognition for MMSE, extended MMSE, and CAMCOG, weighted back to the population.[26,27] It has reported on risk for incident dementia including apoE and ACE.[28–30] It has examined the relationship of cognition to mortality.[18,31] It has reported on the mixed neuropathology found in the brains of the oldest old.[32] In addition, the study has reported on a variety of impairments to healthy life in old age and their population burden.[33–38] The Resource Implication Study has provided data for examination of carer burden and the costs of care for physically and cognitively frail.[3–9,39] The data have been used for projection forward, for these vulnerable groups and also for the costs of long-term care. Liverpool has published on the prevalence of dementia, depression, and neurosis, together with incidence of dementia and schizophrenia.[40–43]

What are the main strengths?

The study is multisite and multidisciplinary. The population is truly representative with high response rates at each stage over diverse sites. Where there is no heterogeneity across sites the study is sufficiently large to provide indicative values for national estimates. The broad scope of measures has allowed the study to contribute to ageing research across a wide range of topics. There are repeat measures on cognition and function, which allows examination of trajectories. There are only two other population-based studies with brain donation in Europe.[44,45] The study weighted the sample towards the over 75 age group at baseline, which has provided more robust data for the oldest old.

What are the main weaknesses?

It would be desirable to have higher response and lower drop out between waves, but analysis can adjust for loss between interviews. Blood taking and clinical assessment (including imaging) at baseline was not possible because of funding constraints, but venepuncture was included at year 6. The risk measures are self-report, using the available validated measures of the era.

Can I get hold of data?

The study actively encourages collaboration, and there are established mechanisms for approaching us via the themes mentioned above (see What does it cover?). Information is available on the website and also through contact with theme leads.

Where can I find out more?

The study website, www.cfas.ac.uk, configures information under themes, documentation, publications, and data. There is also a list of study contacts.

Acknowledgements

We are grateful to our respondents, their families, and their primary care teams. The study has only been possible because of the dedication of a large number of individuals over the years who are listed on the website. The MRC CFA Study has been supported by major awards from the Medical Research Council and the Department of Health. Thanks are also due to the Biological Resource Advisory Group for overseeing this aspect of study and to Lu Gao for preparation of the manuscript.

Downloaded from http://ije.oxfordjournals.org/ at Cambridge University Library on March 12, 2013

For peer review only

For peer review only - http://bmjopen.bmj.com/site/about/guidelines.xhtml

References

1 Saunders PA, Copeland JR, Dewey ME, Larkin BA, Scott A. Alpha: the Liverpool MRC Study of the incidence of dementia and cognitive decline. *Neuroepidemiology* 1992;**11**(suppl. 1):44–47.

2 Chadwick C. The MRC Multicentre Study of Cognitive Function and Ageing: a EURODEM incidence study in progress. *Neuroepidemiology* 1992;**11**(suppl. 1):37–43.

3 Buck D, Gregson BA, Bamford CH *et al*. Psychological distress among informal supporters of frail older people at home and in institutions. The Resource Implications Study Group of the MRC Cognitive Function and Ageing Study. *Int J Geriatr Psychiatry* 1997;**12**:737–44.

4 McNamee P, Gregson BA, Wright K, Buck D, Bamford CH, Bond J. Estimation of a multiproduct cost function for physically frail older people. *Health Econ* 1998;**7**:701–10.

5 McNamee P, Gregson BA, Buck D, Bamford CH, Bond J, Wright K. Costs of formal care for frail older people in England: the resource implications study of the MRC cognitive function and ageing study (RIS MRC CFAS). *Soc Sci Med* 1999;**48**:331–41.

6 Bond J, Farrow G, Gregson BA *et al*. Informal caregiving for frail older people at home and in long-term care institutions: who are the key supporters? *Health Soc Care Community* 1999;**7**:434–44.

7 Melzer D, McWilliams B, Brayne C, Johnson T, Bond J. Profile of disability in elderly people: estimates from a longitudinal population study. *BMJ* 1999;**318**:1108–11.

8 Psychological morbidity among informal caregivers of older people: a 2-year follow-up study. The Resource Implications Study Group of the MRC study of cognitive function and ageing (RIS MRC CFAS). *Psychol Med* 2000;**30**:943–55.

9 McNamee P, Bond J, Buck D. Costs of dementia in England and Wales in the 21st century. *Br J Psychiatry* 2001;**179**:261–66.

10 Solomou W, Richards MPM, Huppert FA, Brayne C, Morgan K. Divorce, current marital status and well-being in an elderly population. *Int J Law, Policy and the Family* 1998;**12**:321–342.

11 Huppert FA, Solomou W, O'Connor S, Morgan K, Sussams P, Brayne C. Aging and lymphocyte subpopulations: whole-blood analysis of immune markers in a large population sample of healthy elderly individuals. *Exp Gerontol* 1998;**33**:593–600.

12 Morgan K, Armstrong GK, Huppert FA, Brayne C, Solomou W. Healthy ageing in urban and rural Britain: a comparison of exercise and diet. *Age Ageing* 2000;**29**:341–48.

13 Huppert FA, Pinto EM, Morgan K, Brayne C. Survival in a population sample is predicted by proportions of lymphocyte subsets. *Mech Ageing Dev* 2003;**124**:449–51.

14 Huppert FA, Pinto E, Morgan K, MRC CFAS, Brayne C. Immune measures which predict 9-year survival in an elderly population sample. *Basic Biology and Clinical Impact of Immunosenescence, Advances in Cell Aging and Gerontology* 2003;**13**:17–28.

15 Pinto EM, Huppert FA, Morgan K, Mrc C, Brayne C. Neutrophil counts, monocyte counts and cardiovascular disease in the elderly. *Exp Gerontol* 2004;**39**:615–19.

16 Rocca WA, Hofman A, Brayne C *et al*. The prevalence of vascular dementia in Europe: facts and fragments from 1980–1990 studies. EURODEM-Prevalence Research Group. *Ann Neurol* 1991;**30**:817–24.

17 Copeland JR, Beekman AT, Braam AW *et al*. Depression among older people in Europe: the EURODEP studies. *World Psychiatry* 2004;**3**:45–49.

18 Matthews FE, Chatfield M, Freeman C, McCracken C, Brayne C. Attrition and bias in the MRC cognitive function and ageing study: an epidemiological investigation. *BMC Public Health* 2004;**4**:12.

19 Matthews FE, Chatfield M, Brayne C. An investigation of whether factors associated with short-term attrition change or persist over ten years: data from the Medical Research Council Cognitive Function and Ageing Study (MRC CFAS). *BMC Public Health* 2006;**6**:185.

20 Copeland JR, Dewey ME, Griffiths-Jones HM. A computerized psychiatric diagnostic system and case nomenclature for elderly subjects: GMS and AGECAT. *Psychol Med* 1986;**16**:89–99.

21 Huppert FA, Brayne C, Gill C, Paykel ES, Beardsall L. CAMCOG–a concise neuropsychological test to assist dementia diagnosis: socio-demographic determinants in an elderly population sample. *Br J Clin Psychol* 1995;**34**:529–41.

22 Mirra SS, Heyman A, McKeel D *et al*. The Consortium to Establish a Registry for Alzheimer's Disease (CERAD). Part II. Standardization of the neuropathologic assessment of Alzheimer's disease. *Neurology* 1991;**41**:479–86.

23 Cognitive function and dementia in six areas of England and Wales: the distribution of MMSE and prevalence of GMS organicity level in the MRC CFA Study. The Medical Research Council Cognitive Function and Ageing Study (MRC CFAS). *Psychol Med* 1998;**28**:319–35.

24 Matthews F, Brayne C. The incidence of dementia in England and Wales: findings from the five identical sites of the MRC CFA Study. *PLoS Med* 2005;**2**:e193.

25 Matthews FE, Dening T. Prevalence of dementia in institutional care. *Lancet* 2002;**360**:225–26.

26 Williams JG, Huppert FA, Matthews FE, Nickson J. Performance and normative values of a concise neuropsychological test (CAMCOG) in an elderly population sample. *Int J Geriatr Psychiatry* 2003;**18**:631–44.

27 Huppert FA, Cabelli ST, Matthews FE. Brief cognitive assessment in a UK population sample—distributional properties and the relationship between the MMSE and an extended mental state examination. *BMC Geriatr* 2005;**5**:7.

28 Yip AG, Brayne C, Matthews FE. Risk factors for incident dementia in England and Wales: The Medical Research Council Cognitive Function and Ageing Study. A population-based nested case-control study. *Age Ageing* 2006;**35**:154–60.

29 Yip AG, Brayne C, Easton D, Rubinsztein DC. An investigation of ACE as a risk factor for dementia and cognitive decline in the general population. *J Med Genet* 2002;**39**:403–06.

30 Yip AG, Brayne C, Easton D, Rubinsztein DC. Apolipoprotein E4 is only a weak predictor of dementia and cognitive decline in the general population. *J Med Genet* 2002;**39**:639–43.

31 Neale R, Brayne C, Johnson AL. Cognition and survival: an exploration in a large multicentre study of the population aged 65 years and over. *Int J Epidemiol* 2001;**30**:1383–88.

32 Pathological correlates of late-onset dementia in a multicentre, community-based population in England and Wales. Neuropathology Group of the Medical Research Council Cognitive Function and Ageing Study (MRC CFAS). *Lancet* 2001;**357**:169–75.

33 Brayne C, Gao L, Matthews F. Challenges in the epidemiological investigation of the relationships between physical activity, obesity, diabetes, dementia and depression. *Neurobiol Aging* 2005;**26**(suppl. 1):6–10.

34 Jagger C, Matthews F. Gender differences in life expectancy free of impairment at older ages. *J Women Aging* 2002;**14**:85–97.

35 McGee MA, Johnson AL, Kay DW. The description of activities of daily living in five centres in England and Wales. Medical Research Council Cognitive Function and Ageing Study. *Age Ageing* 1998;**27**:605–13.

36 Melzer D, McWilliams B, Brayne C, Johnson T, Bond J. Socioeconomic status and the expectation of disability in old age: estimates for England. *J Epidemiol Commun Health* 2000;**54**:286–92.

37 Parker CJ, Morgan K, Dewey ME. Physical illness and disability among elderly people in England and Wales: the Medical Research Council Cognitive Function and Ageing Study. The Analysis Group. *J Epidemiol Commun Health* 1997;**51**:494–501.

38 Spiers NA, Matthews RJ, Jagger C *et al*. Diseases and impairments as risk factors for onset of disability in the older population in England and

For peer review only

Downloaded from http://ije.oxfordjournals.org/ at Cambridge University Library on March 12, 2013

For peer review only - http://bmjopen.bmj.com/site/about/guidelines.xhtml

Wales: findings from the Medical Research Council Cognitive Function and Ageing Study. *J Gerontol A Biol Sci Med Sci* 2005;**60**:248–54.

39 Comas-Herrera A, Wittenberg R, Pickard L, Knapp M, MRC-CFAS. Cognitive impairment in older people: its implications for future demand for services and costs. *Mental Health Research Review* 2001;**8**:37–38.

40 Copeland JR, Chen R, Dewey ME *et al*. Community-based case-control study of depression in older people. Cases and sub-cases from the MRC-ALPHA Study. *Br J Psychiatry* 1999;**175**:340–47.

41 Copeland JR, McCracken CF, Dewey ME *et al*. Undifferentiated dementia, Alzheimer's disease and vascular dementia: age- and gender-related incidence in Liverpool. The MRC-ALPHA Study. *Br J Psychiatry* 1999;**175**:433–38.

42 Copeland JR, Dewey ME, Scott A *et al*. Schizophrenia and delusional disorder in older age: community prevalence, incidence, comorbidity, and outcome. *Schizophr Bull* 1998;**24**:153–61.

43 Saunders PA, Copeland JR, Dewey ME *et al*. The prevalence of dementia, depression and neurosis in later life: the Liverpool MRC-ALPHA Study. *Int J Epidemiol* 1993;**22**:838–47.

44 Polvikoski T, Sulkava R, Myllykangas L *et al*. Prevalence of Alzheimer's disease in very elderly people: a prospective neuropathological study. *Neurology* 2001;**56**:1690–96.

45 Xuereb JH, Brayne C, Dufouil C *et al*. Neuropathological findings in the very old. Results from the first 101 brains of a population-based longitudinal study of dementing disorders. *Ann N Y Acad Sci* 2000;**903**:490–96.

For peer review only

Downloaded from http://ije.oxfordjournals.org/ at Cambridge University Library on March 12, 2013

For peer review only - http://bmjopen.bmj.com/site/about/guidelines.xhtml

STROBE Statement—Checklist of items that should be included in reports of ***cohort studies***

- see below **

	Item No	Recommendation
**Title and abstract**	1	(*a*) Indicate the study's design with a commonly used term in the title or the abstract
		(*b*) Provide in the abstract an informative and balanced summary of what was done and what was found
**Introduction**		
Background/rationale	2	Explain the scientific background and rationale for the investigation being reported
Objectives	3	State specific objectives, including any prespecified hypotheses
**Methods**		
Study design	4	Present key elements of study design early in the paper
Setting	5	Describe the setting, locations, and relevant dates, including periods of recruitment, exposure, follow-up, and data collection
Participants	6	(*a*) Give the eligibility criteria, and the sources and methods of selection of participants. Describe methods of follow-up
		(*b*) For matched studies, give matching criteria and number of exposed and unexposed
Variables	7	Clearly define all outcomes, exposures, predictors, potential confounders, and effect modifiers. Give diagnostic criteria, if applicable
Data sources/ measurement	8*	For each variable of interest, give sources of data and details of methods of assessment (measurement). Describe comparability of assessment methods if there is more than one group
Bias	9	Describe any efforts to address potential sources of bias
Study size	10	Explain how the study size was arrived at
Quantitative variables	11	Explain how quantitative variables were handled in the analyses. If applicable, describe which groupings were chosen and why
Statistical methods	12	(*a*) Describe all statistical methods, including those used to control for confounding
		(*b*) Describe any methods used to examine subgroups and interactions
		(*c*) Explain how missing data were addressed
		(*d*) If applicable, explain how loss to follow-up was addressed
		(*e*) Describe any sensitivity analyses
**Results**		
Participants	13*	(a) Report numbers of individuals at each stage of study—eg numbers potentially eligible, examined for eligibility, confirmed eligible, included in the study, completing follow-up, and analysed
		(b) Give reasons for non-participation at each stage
		(c) Consider use of a flow diagram
Descriptive data	14*	(a) Give characteristics of study participants (eg demographic, clinical, social) and information on exposures and potential confounders
		(b) Indicate number of participants with missing data for each variable of interest
		(c) Summarise follow-up time (eg, average and total amount)
Outcome data	15*	Report numbers of outcome events or summary measures over time
Main results	16	(*a*) Give unadjusted estimates and, if applicable, confounder-adjusted estimates and their precision (eg, 95% confidence interval). Make clear which confounders were adjusted for and why they were included
		(*b*) Report category boundaries when continuous variables were categorized

For peer review only

		(*c*) If relevant, consider translating estimates of relative risk into absolute risk for a meaningful time period
Other analyses	17	Report other analyses done—eg analyses of subgroups and interactions, and sensitivity analyses
**Discussion**		
Key results	18	Summarise key results with reference to study objectives
Limitations	19	Discuss limitations of the study, taking into account sources of potential bias or imprecision. Discuss both direction and magnitude of any potential bias
Interpretation	20	Give a cautious overall interpretation of results considering objectives, limitations, multiplicity of analyses, results from similar studies, and other relevant evidence
Generalisability	21	Discuss the generalisability (external validity) of the study results
**Other information**		
Funding	22	Give the source of funding and the role of the funders for the present study and, if applicable, for the original study on which the present article is based

*Give information separately for exposed and unexposed groups.

Note: An Explanation and Elaboration article discusses each checklist item and gives methodological background and published examples of transparent reporting. The STROBE checklist is best used in conjunction with this article (freely available on the Web sites of PLoS Medicine at http://www.plosmedicine.org/, Annals of Internal Medicine at http://www.annals.org/, and Epidemiology at http://www.epidem.com/). Information on the STROBE Initiative is available at http://www.strobe-statement.org.

STROBE checklist

Item	Reported on page
1(a)	1
1(b)	2
2	3,4
3	4
4	4,5
5	4-7
6 (a)	4,5
7	6
8	5,6
9	6
10	Described elsewhere - see references 1 and 2
11	6
12 (a)	6,7
12 (b)	6,7
12 (c)	6,7
12 (d)	6,7 – described elsewhere see references 1 and 2
12 (e)	N/A
13 (a)	7
13 (b)	7
13(c)	Described elsewhere – see reference 1
14 (a)	7
14(b)	see tables
14 (c)	6

For peer review only - http://bmjopen.bmj.com/site/about/guidelines.xhtml

For peer review only

15 cohort study	N/A
16(a)	see additional table
16(b)	N/A
16 (c)	N/A
17	7/8
18	7/8
19	11
20	9/10
21	9/10
22	13

Reference 1:

Brayne C, McCracken C, Matthews FE. Cohort profile: the Medical Research Council Cognitive Function and Ageing Study (CFAS). *Int J Epidemiol* 2006; 35(5):1140-1145.

Matthews FE, Chatfield M, Freeman C, McCracken C, Brayne C, MRC CFAS (2004), "Attrition and bias in the MRC cognitive function and ageing study: an epidemiological investigation." *BMC Public Health* 4:12

For peer review only

For peer review only - http://bmjopen.bmj.com/site/about/guidelines.xhtml
